# Profiling and Bioactivity of Polyphenols from the Omani Medicinal Plant *Terminalia dhofarica* (syn. *Anogeissus dhofarica*)

**DOI:** 10.3390/molecules30040952

**Published:** 2025-02-18

**Authors:** Jonas Kappen, Luay Rashan, Katrin Franke, Ludger A. Wessjohann

**Affiliations:** 1 Department of Bioorganic Chemistry, Leibniz Institute of Plant Biochemistry (IPB), 06120 Halle (Saale), Germany; jkappen@ipb-halle.de; 2 Biodiversity Unit, Research Center, Dhofar University, Salalah 211, Oman; lrashan@du.edu.om; 3 Institute of Chemistry, Martin Luther University Halle-Wittenberg, 06120 Halle (Saale), Germany

**Keywords:** *Terminalia dhofarica*, *Anogeissus*, phytochemical profiling, UHPLC-HRMS, NMR, structure elucidation, antibiotic, antifungal

## Abstract

Several polyphenol-rich *Terminalia* species (Combretaceae) are known to accelerate wound healing. Recently, the Omani medicinal plant *Anogeissus dhofarica* (now *Terminalia dhofarica*) was attributed to the genus *Terminalia* based on phylogenetic studies. Leaves, bark, and extracts of *T. dhofarica* are traditionally used for various medicinal purposes, including wound treatment and personal hygiene. In the present study, the phytochemical profile of leaves from *T. dhofarica* was evaluated by ultra-high-performance liquid chromatography coupled with electrospray ionization high-resolution mass spectrometry (UHPLC-ESI-HRMS) and nuclear magnetic resonance (NMR) spectroscopy. Simple phenolics, polyphenolics (e.g., flavonoids and tannins) and their glucosides were characterized as major metabolite classes. In addition, 20 phenolics were isolated and structurally identified. Nine of these compounds were never described before for *T. dhofarica*. For the first time, we provide complete NMR data for 1-*O*-galloyl-6-*O*-*p*-coumaroyl-d-glucose (**1**). Biological screening demonstrated moderate efficacy against the Gram-negative bacterium *Aliivibrio fischeri,* the phytopathogenic fungus *Septoria tritici,* and the oomycete *Phytophthora infestans*. In summary, the data expand the knowledge of the phytochemistry of the underexplored species *T. dhofarica* and underscore its potential for therapeutic applications, particularly in the context of traditional medicine.

## 1. Introduction

*Terminalia dhofarica* (A.J.Scott) Gere & Boatwr. (formerly referred to by the homotypic synonym *Anogeissus dhofarica* A.J.Scott) belongs to the Combretaceae family and is an endemic species of the Dhofar region in Oman and southeastern Yemen, thriving in monsoon ecosystems [1]. Previously integrated in the genus *Anogeissus*, it was transferred with the whole genus *Anogeissus* into the genus *Terminalia* in 2017 [2,3,4], which led to formal taxonomic name changes.

The former genus *Anogeissus* is primarily distributed across southern Asia, the Arabian Peninsula, and West Africa [5]. Many former *Anogeissus* species have significant ethnomedicinal uses ranging from gastric disorders, skin diseases, and diabetes to wound healing and coughs [6,7,8,9]. The bioactivity is primarily attributed to their high content of phenolic compounds such as gallic acid, ellagic acid, and their derivatives, as well as flavonoids like quercetin and rutin [10,11].

Traditionally, leaves, bark, and extracts of *T. dhofarica* are used for various medicinal purposes, including wound treatment and as antiseptic in personal hygiene [8,12]. Previous studies showed that aqueous and alcoholic extracts with a high phenolic content exhibit potent antioxidant activity and display antibacterial and antifungal activities [8,13]. Despite its traditional use and promising activities, the phytochemical and pharmacological profile of *T. dhofarica* remained underexplored compared to other species of the genus [10]. Recent investigations by Maqsood et al. [14] and Abuarqoub et al. [15] led to a tentative annotation of 28 compounds, predominantly flavonoids and phenolic acids. These results are in line with the observed strong antioxidant and radical scavenging properties. Additionally, the extracts showed potential anticancer, antidiabetic, and anti-inflammatory activities, promoted fibroblast migration and enhanced wound healing, which confirmed its traditional medicinal uses [14,15].

The current study represents the first comprehensive phytochemical characterization of the species. Methanolic crude extracts from leaves were analyzed by UHPLC-ESI-HRMS and NMR for major metabolites and screened for antibacterial and antifungal activity. The identity of constituents was verified by isolation, characterization and complete structure elucidation based on extensive spectroscopic methods.

## 2. Results and Discussion

### 2.1. Metabolite Profiling

**Figure 1:**
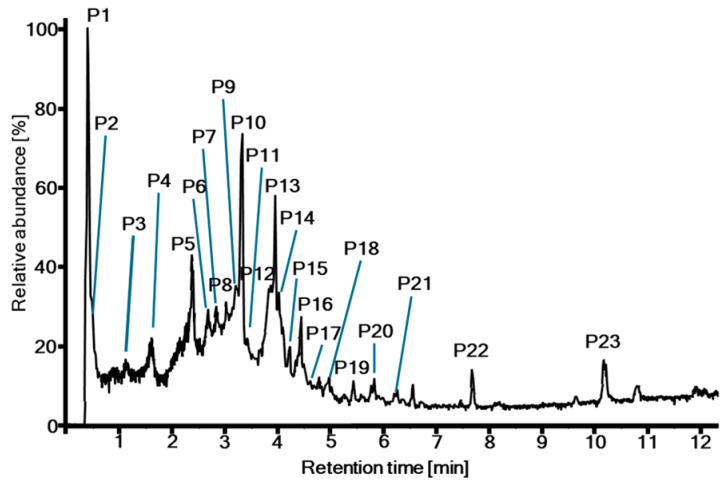
UHPLC-ESI-HRMS total ion chromatogram of the crude extract from *T. dhofarica* leaves.

**Figure 2:**
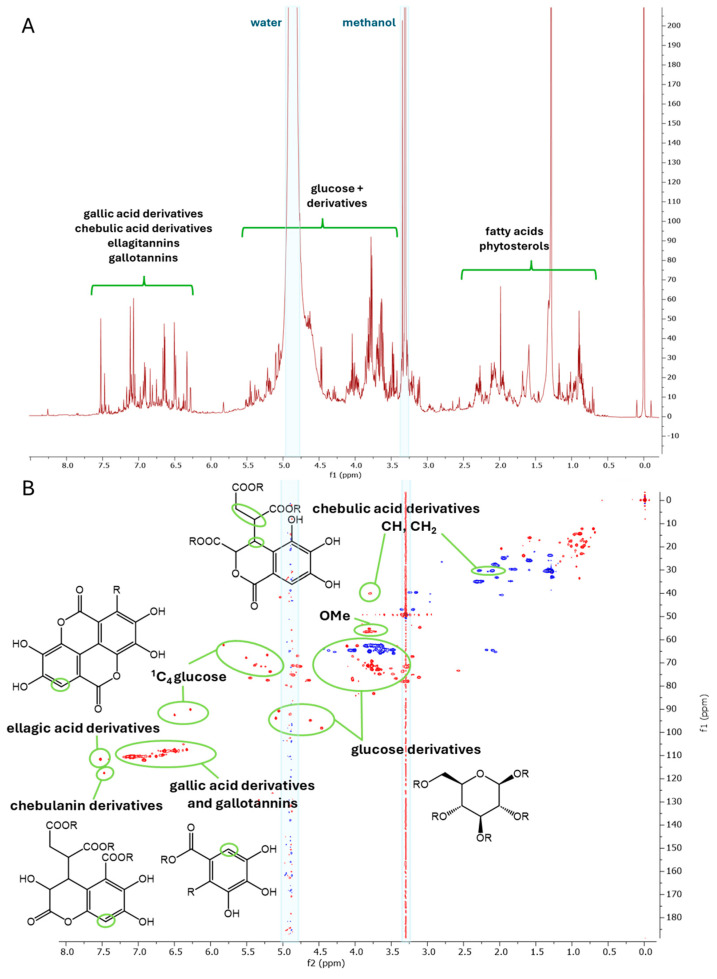
NMR metabolite profiling (Methanol-*d*_4_, 600/150 MHz) of the crude extract from *T. dhofarica* leaves; (**A**) ^1^H NMR; (**B**) HSQC with annotation of characteristic ^1^*J*_CH_ correlations.

Dried leaves from *T. dhofarica* were pulverized and exhaustively extracted with 80% methanol to yield a crude extract. This extract was screened for antibacterial and antifungal activity (Figures S1–S4, see Section 2.4). Furthermore, the metabolite profile was analyzed using UHPLC-ESI-HRMS (Figure 1 and Figure S5, Table 1). An aliquot of the powder was extracted with deuterated methanol and subjected to NMR analysis (Figure 2).

**Figure 3:**
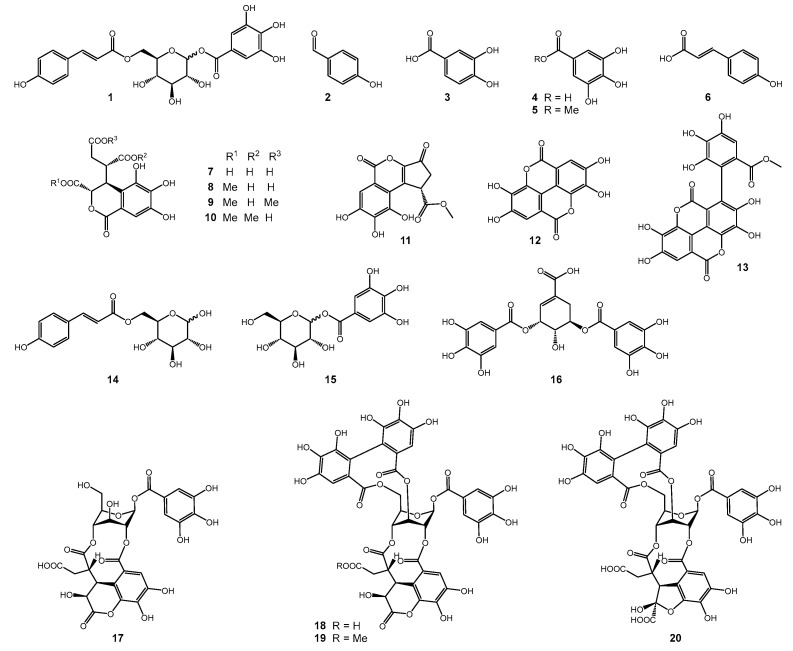
Structures of compounds isolated from the leaf extract of *T. dhofarica*.

The NMR screening of the crude extract revealed a profile consistent with the known characteristics of the genus [10]. Many intense singlet signals appear in the aromatic region of the ^1^H spectrum, between 6.2 and 7.5 ppm (Figure 2A). The HSQC spectrum associates these signals with a consistent ^13^C shift of approximately 110 ppm (Figure 2B) in accordance with signals for gallic acid and its derivatives, such as ellagic acid or follow-up tannin structures. In addition to the expected signals for fatty acids, sterols and sugars (Figure 2A), several distinct signals were identified that, according to literature reports, could be attributed to derivatives with a chebulic acid core (Figure 2B). Some compounds contain structural features of both groups, gallic acid derivatives and chebulic acid derivatives, as, e.g., chebulagic acid (**18**, Figure 3).

The presence of gallic acid, chebulic acid and diverse derivatives was further confirmed through UHPLC-ESI-HRMS analysis, which provided a more detailed view of the complexity and diversity of metabolites (Table 1, Figure 1 and Figure S5). A total of 32 metabolites were preliminary annotated from the total ion chromatogram (TIC) of the crude extract, although in some cases, compounds eluted simultaneously. The majority of signals could be attributed to phenolic acids (Table 1, P1 gallic acid + quinic acid; P3 protocatechuic acid; P11 p-coumaric acid), tannins (Table 1, P2; P3 terflavin B, *O*-galloyl punicalin; P4; P5; P6 brevifolincarboxylate; P7 *O*-galloyl bis-*O*-HHDP-glucose; P8; P9; P10; P12; P13; P14, P16, P17, P18 *O*-methyl ellagic acid, P21, P22), or flavonoids (Table 1, P7 gallocatechin, P15; P18 galloylvitexin isomers; P19; P20), following the classification of compounds by Singh et al. [10]. Consequently, *T. dhofarica* appears to possess a metabolic profile closely resembling that of its relatives within the genus [10,14,15].

Our data partly overlapped with previous studies on the composition of the crude extract of *T. dhofarica*. Abuarqoub et al. [15] identified seven phenolic acids by retention times of standard substance only. Two of these acids were also found within this study, both in the LC-HRMS screening and in the isolation approach (gallic acid (**4**), P1; protocatechuic acid (**3**), P3). Noteworthy, Abuarqoub et al. identified *ortho*- and *meta*- but no *para*-coumaric acid which, however, is the only coumaric acid derivative found in this study (**6**, P11). Maqsood et al. [14] annotated 23 compounds by HRMS data from the crude extract. However, their tentative assignments were based on molecular formula calculations with excessively high errors ranging from −299 ppm to +780 ppm [14]. This reduces the reliability of their results significantly since mass accuracy should be lower than ±10 ppm. The authors did not provide MS fragmentation data that could support the annotation. Nevertheless, eight of these compounds were also found in this study. Remarkably, chebulagic acid (**18**, P13), the main compound from this study, was not described by Maqsood et al., although they annotated chebulic acid (**7**, P2) on one hand and corillagin (P9) on the other hand which are the two parts of chebulagic acid.

### 2.2. Isolation and Structure Elucidation

The separation of the crude extract by liquid–liquid partition followed by different chromatographic techniques resulted in the isolation of 20 phenolic compounds (Figure 3). All compounds were identified by extensive spectral analysis (HRMS, NMR) and comparison with previously reported data from the literature [16,17,18,19,20,21,22,23,24,25,26,27,28,29,30,31,32,33,34,35,36]. A full spectroscopic data set for compounds **2**–**20** can be found in the Supporting Information. Fifteen of the 20 isolated compounds were never described before for *T. dhofarica*.

Compound **1** (1-*O*-galloyl-6-*O*-*p*-coumaroyl-d-glucose), a twice modified glucose molecule with gallic acid at position 1 and *p*-coumaric acid at position 6, was postulated by Mei et al. as a constituent of a Chinese herbal preparation based on extensive HRMS fragmentation analysis only [16]. Here, we provide for the first time complete NMR data for compound **1**. Notably, it was obtained as a mixture of *α-* and *β*-d-glucoside.

**Figure 4:**
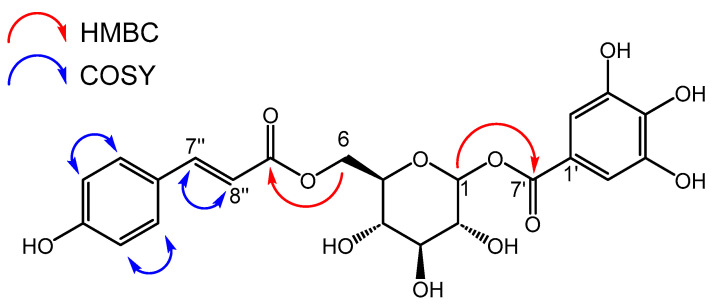
Structure of compound **1** with crucial NMR correlations.

Compound **1** (Figure 4) was isolated as a white solid. The molecular formula was identified as C_22_H_22_O_12_ by its negative ion at *m/z* 477.1030 [M–H]^−^ (calcd. for 477.1038 C_22_H_21_O_11_^−^) in the ESI-HRMS spectrum. All NMR data, as well as 2D correlations, are presented in Table 2. The ^1^H spectra (Figure S6) showed five aromatic signals. The presence of a galloyl group was deduced from HSQC (Figure S9) and HMBC correlations (Figure S10) of the singlet at δ_H_ 7.13 (2 H, *s*, H-2′ + H-6′), resulting in the annotation of the ^13^C signals δ_C_ 110.5, 120.5, 140.7, 146.6 and 166.9 to this substructure. Two pairs of coupling aromatic protons at δ_H_ 7.63 and 6.36 (each 1 H, *d*, *J* = 15.7 Hz, H-7″ + H-8″) and at δ_H_ 7.45 and 6.80 (each 2 H, *d*, *J* = 8.5 Hz, H-2″ and H-6″ + H-3″ and H-5″) indicated the presence of a *trans* configurated double bond and a *para*-substituted benzene ring, respectively. In combination with the corresponding nine carbons, these signals were attributed to a coumaroyl group. The pattern of aliphatic proton signals, including two anomeric protons at δ_H_ 5.66 (0.5 H, *d*, *J* = 7.7 Hz, H-1*β*) and 5.66 (0.5 H, *d*, *J* = 3.3 Hz, H-1*α*), three multiplet signals at δ_H_ 3.41–3.46, 3.46–3.52, and 3.65–3.70 and signals of a CH_2_ group at δ_H_ 4.31 (1 H, *dd*, *J* = 5.6, 12.2 Hz, H-6b) and δ_H_ 4.50 (1 H, *dd*, *J* = 2.8, 12.2 Hz, H-6a), suggested the presence of one glucopyranosyl moiety. This was further supported by strong COSY correlations (Figure S8) and the HSQC correlation of the aliphatic proton signals (Table 2). The specific connection pattern of all three moieties was determined by HMBC correlation of the glucose protons H-1 to the galloyl carbon C-7′ and of H-6a and H-6b to the coumaroyl carbon C-9″ (Table 2, Figure S10). Therefore, the compound is identified as 1-*O*-galloyl-6-*O*-coumaroyl-d-glucose. In the glucose moiety, α and β configurations appeared in a ratio of 1:1 by comparison of integrals in the ^1^H spectrum. Structurally, compound **1** is close to fishertannin F (1-*O*-galloyl-6-*O*-feruloyl-*β*-d-glucose) [37], with an additional methoxy group in the cinnamic acid core. Consequently, the NMR data are mainly in accordance with those reported by Zhang et al. [37].

Compounds **14** (6-*O-trans*-*p*-coumaroyl-d-glucopyranose) and **15** (1-*O*-galloyl-d-glucose) represent substructures of **1,** and both anomeric configurations appeared. This is a well-reported phenomenon for **14** [32].

The anomeric protons of the sugar moieties of compounds **17**–**20** show unusual chemical shifts and small coupling constants (e.g., 6.35, 1 H, *d*, *J* = 2.8 Hz, for H-1 in **17**). Usually, *β*-glucose appears in the energetically favored ^4^C_1_ chair conformation. However, in ellagitannins with bridging 2,4-*O*-chebuloyl substituents, the *β*-glucose ring is locked into the inverted ^1^C_4_ conformation with all ring protons in equatorial instead of axial positions, resulting in small vicinal couplings (<4 Hz) [35].

### 2.3. Evaluation of Artifacts

**Figure 5:**
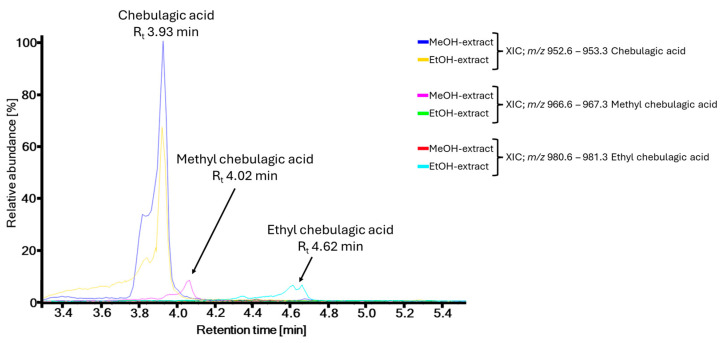
Test for artifacts on the example of chebulagic acid and methyl chebulagic acid by UHPLC-ESI-HRMS analysis of methanolic and ethanolic crude leaf extracts of *T. dhofarica*.

Remarkably, some of the isolated compounds exhibited methylations of carboxyl functional groups (**5**, **8**, **9**, **10**, **11**, **13**, and **19**). However, these compounds were absent in the UHPLC-ESI-HRMS analysis of the crude extract, but their unmethylated form was detected, such as chebulic acid (**7**) (Table 1, P2), brevifolincarboxylate (Table 1, P6), or flavogallonate (Table 1, P8). Since methanol was used to extract the plant material and as a solvent in multiple purification steps, the mentioned compounds may be artifacts of the isolation process rather than true metabolites of *T. dhofarica*. To investigate this possibility, a small-scale extraction of leaves was performed with methanol versus ethanol. The analysis of both total ion chromatograms (TICs) revealed all the above-mentioned compounds as probable artifacts. Exemplary, Figure 5 presents the extracted ion chromatogram (XIC) of the methanolic and ethanolic extracts, filtered for pure chebulagic acid and its methylated and ethylated derivatives. The pure compound is present in both extracts, confirming it as a true metabolite. However, the methylated derivative appears only in the methanolic extract, and the ethylated derivative solely in the ethanolic extract, strongly suggesting both are artifacts. This finding is particularly noteworthy, as methylated chebulagic acid was also reported as an artifact in the isolation of *Terminalia chebula* Retz., another species from the genus [2,38]. In contrast, true methylated metabolites, such as methylated derivatives of ellagic acid (Table 1, P18, P21, and P22), were confirmed by UHPLC-ESI-HRMS analysis in both extracts. Thus, to the best of our knowledge, 15 of 20 isolated compounds are directly of plant origin, and from these, 9 compounds (**1**; **2**; **6**; **14**–**18**; **20**) are described for the first time within this species.

### 2.4. Evaluation of Bioactivity

The methanolic crude extract of leaves was screened for antibacterial and antifungal activity (Table 3, Figures S1–S4). In our investigation, the extract exhibited moderate activity against Gram-negative bacteria (*Aliivibrio fischeri* at a concentration of 500 µg/mL, Figure S1) but showed no activity against Gram-positive bacteria (*Bacillus subtilis*). In antifungal assays, the crude extract also exhibited moderate activity against the phytopathogenic fungus *Septoria tritici* and the oomycete *Phytophthora infestans* (around 80% of inhibition at 100 and 10 µg/mL, respectively) but did not influence the growth of *Botrytis cinerea*.

In general, *T. dhofarica* seems not to deliver consistent effects in antibacterial assays. Maqsood et al. reported no activity against Gram-negative bacteria (*E. hormaechei*) but observed significant inhibition of Gram-positive bacteria (*S. aureus*) in a ZOI assay [14]. Conversely, Marwah et al. reported substantial activity against Gram-positive bacteria (*S. aureus* at 250 µg/mL) and moderate activity against Gram-negative bacteria (*P. aeruginosa* at 500 µg/mL) [8]. This might be due to differences in assay methods, bacterial strains, or the choice of plant material and extraction conditions (see above for artifact formation). Notably, while both previous studies used mixed plant material for extraction, this analysis focused exclusively on leaves.

Based on the biological effects of the crude extract, all isolated compounds, including the potential artifacts, were screened for biological activity against the Gram-negative bacterium *A. fischeri*, as well as the phytopathogenic fungi *B. cinerea*, *S. tritici* and the oomycete *P. infestans* (Table 3). In contrast to expectations, none of the isolated compounds exhibited inhibitory effects on *A. fischeri*, although the crude extract displayed moderate activity (Figure S1). This suggests that the active compound was either not isolated or modified (e.g., by methylation) or that synergistic effects were required, which may have been lost during the separation of synergistic partners.

Several compounds demonstrated some level of antifungal activity against *S. tritici* and *P. infestans* (up to 90% inhibition at 100 µM, Figures S2–S4). The most remarkable effect can be reported for the artifact 6′-*O*-methyl-chebulagic acid (**19**), which showed 82% inhibition against *P. infestans* at a concentration of 10 µM (Figure S2). In general, the methylated artifacts were most active against this oomycete. Thus, the reported antifungal properties of *T. dhofarica* may be attributed to the combined effects of various active polyphenolic compounds with rather nonspecific activity. This is common for mixtures of plant phenolics. It is in alignment with the use of crude mixtures in external (or intestinal) applications, as the tanning and gluing effect underlying these compounds on microorganisms is not systemic.

## 3. Materials and Methods

### 3.1. Materials and Chemicals

TLC plates: silica gel 60 normal phase (SG60), silica gel 60 reversed phase 18 F_254_ (Merck, Darmstadt, Germany) or silica gel 60 reversed phase 2 UV_254_ (Macherey-Nagel, Düren, Germany); Column materials: Lichroprep RP 18 40–63 µm (Merck, Darmstadt; Germany), Sephadex LH 20 (GE Healthcare, Uppsala, Sweden), Sephadex G10 (Pharmacia, Uppsala, Sweden); Solvents: ultrapure water (ThermoScientific Barnstead GenPure Pro, Langenselbold, Germany), methanol, ethyl acetate (technical grade solvents distilled prior use), *n*-heptane (Roth, Karlruhe, Germany), acetonitrile for LC-MS Chromasolv, formic acid for mass spectrometry (Honeywell Fluka, Seelze, Germany); DMSO (Duchefa Biochemie, Haarlem, The Netherlands); NMR solvents: methanol-*d*_4_, DMSO-*d*_6_ (Deutero, Kastellaun, Germany); Chemicals: vanillin (Tokyo Chemicals, Tokyo, Japan), chloramphenicol (Roth, Karlsruhe, Germany), 2-aminoethyl diphenylborinate, epoxiconazole and terbinafine (Sigma-Aldrich, Darmstadt, Germany).

### 3.2. Analytical Instruments and General Procedures

Thin layer chromatography (TLC) analyses were performed using different solvent systems, as indicated in Section 3.4. To visualize the compound spots, long-wavelength UV light (366 nm), short-wavelength UV light (254 nm) and spraying with vanillin–H_2_SO_4_ reagent, followed by heating or spraying with natural product spray reagent (1 g 2-aminoethyl diphenylborinate/200 mL methanol) were applied.

Low-resolution ESI-MS spectra were performed on a Sciex API-3200 instrument (Applied Biosystems, Concord, ON, Canada) combined with an HTC-XT autosampler (CTC Analytics, Zwingen, Switzerland).

The semi-preparative HPLC was performed on a Shimadzu prominence system (Kyoto, Japan), which consists of an SPD-M20A diode array detector, an FRC-10A fraction collector, a CBM-20A communications bus module, a DGU-20A5R degassing unit, an LC-20AT liquid chromatograph, and a SIL-20A HT autosampler.

The UHPLC-ESI-HRMS spectra were acquired using a TripleTOF (time of flight) 6600-1 mass spectrometer (Sciex, Darmstadt, Germany) combined with an ACQUITY UPLC I-Class UHPLC System (Waters GmbH, Eschborn, Germany) as described by Kappen et al. [39] with minor modifications. For separation, a Waters Acquity UPLC^®^ BEH C18 column (1.7 µm, 130 Å, 50 × 2.1 mm I.D., Waters GmbH, Eschborn, Germany) was used. Data acquisition was performed in MS1-TOF mode in a mass range of *m*/*z* 65 to 1250 with an accumulation time of 75 ms and in MS2-TOF mode in the *m*/*z* range of 50–1000 with an accumulation time of 20 ms.

^1^H and ^13^C NMR spectra were recorded on an Agilent DD2 400 NMR spectrometer (Santa Clara, CA, USA) at 399.917 and 100.570 MHz, respectively. Chemical shifts are reported relative to TMS (^1^H NMR) or peaks of solvent. For samples with low concentration, 1D ^1^H and ^13^C NMR spectra and 2D spectra (HSQC, HMBC, COSY, TOCSY, NOESY) were recorded on a Bruker Avance Neo 500 NMR spectrometer (Billerica, MA, USA) at 500.234 and 125.797 MHz, respectively, using a 5 mm prodigy probe with the TopSpin 4.0.7 spectrometer software or on an Agilent VNMRS 600 MHz NMR spectrometer equipped with 5 mm inverse detection cryoprobe, using standard CHEMPACK 8.1 pulse sequences implemented in Varian VNMRJ 4.2 spectrometer software.

### 3.3. Plant Material

Leaves of *Terminalia dhofarica* (A.J.Scott) Gere & Boatwr. (synonym *Anogeissus dhofarica* A.J.Scott) were collected in autumn 2019 and 2020 in Wadi Nahiz, which is located on the northern side of Salalah City, Dhofar, Sultanate of Oman. The leaves were shadow-dried at room temperature, pulverized, and stored at room temperature. A voucher (ADA/11/2020) was deposited in the herbarium of the Natural & Medical Sciences Research Center, University of Nizwa, Oman.

### 3.4. Isolation

Dried pulverized leaves (300 g) from T. *dhofarica* were exhaustively extracted with 80% aq. methanol to produce 92 g of dried crude extract after evaporation of the solvent. An aliquot of the crude extract (30.8 g) was successively partitioned by liquid–liquid extraction between water (700 mL) and *n*-heptane (2 × 250 mL), followed by ethyl acetate (6 × 300 mL). This resulted in three fractions: *n*-heptane (1.6 g), ethyl acetate (4.2 g), and water (20.8 g).

The ethyl acetate fraction was submitted to an RP18 column (l: 34 cm, d: 3.5 cm) and eluted with a mixture of methanol and water (1:1, *v*/*v*), which yielded three fractions (A1–A3), based on the TLC profile (RP18, MeOH/H_2_O, 1:1, *v*/*v*) of which A1 (Rf 0.95–0.59) and A2 (Rf 0.59–0.38) were further purified. A3 was identified as ellagic acid (**12**, 547.4 mg, Rf = 0.36 in MeOH/H_2_O (1:1, *v*/*v*) on RP18.

A1 was submitted to a Sephadex G10 column (l: 120 cm, d: 3.5 cm) with a mixture of methanol and water (1:4, *v*/*v*), yielding seven fractions (B1–B7), based on the TLC profile (RP18, MeOH/H_2_O, 2:3, *v*/*v*), of which B2 (Rf 0.98–0.88), B5 (Rf 0.79–0.62), and B6 (Rf 0.62–0.31) were further purified. B3 (Rf 0.83) was identified as gallic acid (**4**), 153.8 mg, Rf = 0.83 in MeOH/H_2_O (2:3, *v*/*v*) on RP18.

B2 was purified by preparative reversed-phase HPLC (Agilent-Zorbax Eclipse-XDB C18, 5 µm, 9.4 mm × 250 mm) using a water + 0.1% formic acid (A) and methanol + 0.1% formic acid (B) gradient system (0−3.0 min, 5% B; 3.0–43.0 min, 5–25% B) and a flow rate of 1.50 mL/min at 25 °C to yield chebulic acid (**7**) (4.6 mg, Rt = 8.80 min), 1-*O*-galloyl-d-glucose (**15**) (2.5 mg, Rt = 12.48 min), protocatechuic acid (**3**) (3.5 mg, Rt = 26.71 min), 12-*O*-methyl chebulic acid (**8**) (8.9 mg, Rt = 28.93 min), 11,12-*O*-dimethyl chebulic acid (**9**) (4.1 mg, Rt = 38.07 min), and 12,13-*O*-dimethyl chebulic acid (**10**) (6.5 mg, Rt = 41.40 min).

B5 was submitted to an RP18 column (l: 36 cm, d: 3.5 cm) with a gradient of methanol and water (500 mL, 1:4, *v*/*v*; 500 mL, 1:2, *v*/*v*; 500 mL, 2:3, *v*/*v*), yielding nine fractions (C1–C9), based on the TLC profile (RP18, MeOH/H_2_O, 1:1, *v*/*v*), of which C4 (Rf 0.72) and C7 (Rf 0.70–0.54) were further purified.

C4 was submitted to a Sephadex LH20 column (l: 60 cm, d: 2.5 cm) with methanol yielding eight fractions (D1–D8), of which D3 was identified as chebulagic acid (**18**) (16.5 mg, Rf = 0.57 in MeOH/H_2_O (2:3, *v*/*v*) on RP18.

C7 was purified by preparative reversed-phase HPLC (YMC-Triart C18, 5 µm, 10 mm × 150 mm) using a water + 0.1% formic acid (A) and methanol + 0.1% formic acid (B) gradient system (0–2.5 min, 35% B; 2.5–22.5 min, 35–50% B; 22.5–27.5 min, 50–60% B) and a flow rate of 2.64 mL/min at 25 °C to yield 7″-*O*-methyl flavogallonate (**13**) (2.1 mg, Rt = 11.50 min), and 11-*O*-methyl brevifolincarboxylate (**11**) (1.5 mg, Rt = 13.80 min).

B6 was submitted to an RP18 column (l: 36 cm, d: 3.5 cm) and eluted with a 10% step gradient of methanol and water with 0.1% TFA (10–40% MeOH, each 250 mL; 50–90% MeOH, each 200 mL; 100% MeOH, 400 mL) which yielded thirteen fractions (E1–E13), based on the TLC profile (RP18, MeOH/H_2_O, 2:3, *v*/*v*) of which E8 (Rf 0.63–0.25) was further purified.

E8 was purified by preparative reversed-phase HPLC (Agilent-Zorbax Eclipse-XDB C18, 5 µm, 9.4 mm × 250 mm) using a water + 0.1% formic acid (A) and methanol + 0.1% formic acid (B) gradient system (0–6.0 min, 25% B; 6.0–36.0 min, 25–45% B; 36.0–37.0 min, 45–100%) and a flow rate of 3.3 mL/min at 25 °C to yield phyllanembilinin C (**20**) (1.2 mg, Rt = 11.62 min), chebulanin (**17**) (2.4 mg, Rt = 15.92 min), 3,5-di-*O*-galloylshikimic acid (**16**) (1.4 mg, Rt = 18.38 min), and 6′-*O*-methyl-chebulagic acid (**20**) (4.8 mg, Rt = 21.74 min).

A2 was submitted to a Sephadex LH20 column (l: 75 cm, d: 2.5 cm) with methanol, yielding five fractions (F1–F5), based on the TLC profile (RP18, MeOH/H_2_O, 1:1, *v*/*v*), of which F1 (Rf 0.62), F2 (Rf 0.55), and F4 (Rf 0.36–0.24) were further purified. F3 (Rf 0.53) was identified as 7-*O*-methyl gallic acid (**5**) (15.2 mg, Rf = 0.53 in MeOH/H_2_O (1:1, *v*/*v*) on RP18).

F1 was purified by preparative reversed-phase HPLC (YMC-ODS-A C18, 12 µm, 10.0 mm × 150 mm) using a water + 0.1% formic acid (A) and methanol + 0.1% formic acid (B) gradient system (0–2.5 min, 30% B; 2.5–17.5 min, 30–45% B) and a flow rate of 3.6 mL/min at 25 °C to yield *trans*-*p*-coumaric acid (**6**) (3.3 mg, Rt = 8.41 min).

F2 was purified by preparative reversed-phase HPLC (Merck-LiChrospher C18, 5 µm, 10.0 mm × 250 mm) using a water (A) and acetonitrile (B) gradient system (0–3.5 min, 12% B; 3.5–18.5 min, 12–20% B) and a flow rate of 4.00 mL/min at 25 °C to yield *p*-hydroxybenzaldehyde (**2**) (2.9 mg, Rt = 15.89 min).

F4 was purified by analytical reversed-phase HPLC (YMC-ODS-A C18, 12 µm, 10.0 mm × 150 mm) using a water (A) and methanol (B) gradient system (0–2.5 min, 20% B; 2.5–20.0 min, 20–38% B) and a flow rate of 4.80 mL/min at 25 °C to yield 6-*O*-*trans*-*p*-coumaroyl-*β*-d-glucopyranose (**14**) (0.9 mg, Rt = 9.45 min), and 1-*O*-galloyl-6-*O*-*trans*-*p*-coumaroyl-*β*-d-glucopyranose (**1**) (0.6 mg, Rt = 19.97 min).

### 3.5. Biological Assays

Antibacterial assays: The crude extract and compounds were evaluated against the Gram-negative *Aliivibrio fischeri* (DSM507) using bioluminescence, following the method as outlined by Ware et al. (2023) [40], and against the Gram-positive *Bacillus subtilis* 168 (DSM 10) using absorption measurements as described by Kappen et al. [39]. In both assays, the synthetic bacteriostatic antibiotic chloramphenicol (100 µM) was applied as a positive control to achieve complete bacterial growth inhibition (100%). The results (mean ± standard deviation, n = 6) are presented as relative values (percent inhibition) compared to the negative control (bacterial growth without test compound). Negative values indicate an increase in bacterial growth.

Antifungal assays: The antifungal activity was tested in triplicate on the phytopathogenic ascomycetes *Botrytis cinerea* Pers. and *Septoria tritici* Desm. and the oomycete *Phytophthora infestans* (Mont.) de Bary according to protocols from the Fungicide Resistance Action Committee (FRAC) with minor modifications as described by Ware et al. [40]. The commercially used fungicides, epoxiconazole and terbinafine (Sigma-Aldrich, Darmstadt, Germany), served as positive control.

## 4. Conclusions

In conclusion, the Omani medicinal plant *Terminalia dhofarica* was found to be very rich in phenolic acids, tannins, and flavonoids, as well as their glucosides, as major compound classes. This is consistent with other species in the genus. A total of 20 compounds were isolated, including the first full characterization of compound **1** with a complete set of NMR data to unequivocally determine its structure. However, a critical examination of the data revealed that seven compounds isolated are likely artifacts of the isolation process due to the methylation of carboxyl groups. Therefore, 13 compounds remain with true plant origins, of which 9 were described for the first time within this species. All metabolites detected or isolated represent phenols or polyphenols. This compound class is known for its wound-healing effects based on anti-inflammatory, antimicrobial, and antioxidant properties [41]. The polyphenol content of *T. dhofarica* is also in line with the moderate antibacterial and antifungal effects observed within this study. In summary, our data corroborate the reported non-systemic use of *T. dhofarica* extracts and underline the traditional application of the species in wound treatment and as antiseptics.

## Figures and Tables

**Table 1:** Peak list of the UHPLC-ESI-HRMS analysis of the crude extract from *T. dhofarica* leaves.

Peak No. *	t_R_ (min)	*m*/*z* [M − H]^−^ Measured	Error (ppm)	Molecular Formula	MS^2^ Product Ions *m*/*z* (rel. Intensity [%])	Annotation
P1	0.38	169.0151	5.1	C_7_H_6_O_5_	169 (28), 125 (100), 81 (6), 79 (22), 69 (6)	Gallic acid (**4**)
		191.0562	0.5	C_7_H_12_O_6_	191 (100), 93 (5), 85 (12)	Quinic acid
P2	0.52	355.0310	0.9	C_14_H_12_O_11_	355 (31), 337 (100), 293 (6), 249 (29), 205 (50), 193 (40), 187 (8), 179 (22), 163 (26), 149 (18), 135 (5)	Chebulic acid (**7**)
		331.0675	1.3	C_13_H_16_O_10_	331 (100), 271 (24), 211 (18), 169 (86), 151 (11), 125 (26), 123 (20), 107 (10)	Galloylglucoside (e.g., **15**)
P3	1.04	153.0191	−1.5	C_7_H_6_O_4_	153 (2), 109 (100), 91 (10), 81 (7), 65 (9)	Protocatechuic acid (**3**)
		783.0649	−4.8	C_34_H_24_O_22_	783 (100), 631 (3), 481 (4), 451 (16), 301 (53), 275 (14), 257 (3)	Terflavin B
		933.0596	−4.7	C_41_H_26_O_26_	933 (100), 781 (10), 721 (8), 601 (13), 575 (4), 451 (4), 299 (4)	*O*-Galloyl punicalin
P4	1.58	1083.0570	−2.1	C_48_H_28_O_30_	1083 (100), 781 (9), 721 (3), 601 (15), 575 (5), 301 (3) 273 (2)	Punicalagin isomer I
P5	2.36	1083.0553	−3.7	C_48_H_28_O_30_	1083 (100), 781 (10), 721 (2), 601 (18), 575 (5), 301 (2) 273 (2)	Punicalagin isomer II
P6	2.63	291.0146	−0.1	C_13_H_8_O_8_	291 (6), 247 (100), 219 (8), 191 (19), 173 (10), 145 (17)	Brevifolincarboxylate
		121.0294	−0.9	C_7_H_6_O_2_	121 (86), 92 (100), 65 (3)	Hydroxybenzaldehyde (**2**)
P7	2.82	935.0763	−3.5	C_41_H_28_O_26_	935 (100), 633 (9), 301 (5), 275 (17)	*O*-Galloyl bis-HHDP-glc
		305.0693	8.6	C_15_H_14_O_7_	305 (60), 225 (34), 97 (100), 80 (16)	Gallocatechin
P8	3.01	651.0823	−2.5	C_27_H_24_O_19_	651 (98), 633 (9), 481 (44), 275 (26), 247 (12), 231 (18), 205 (14), 203 (15), 169 (100), 125 (29)	Chebulanin (**17**)
		469.0039	−2.1	C_21_H_10_O_13_	425 (27), 301 (100), 299 (27), 282 (9), 271 (14), 244 (9), 228 (8), 216 (8), 200 (6), 172 (12), 144 (6)	Flavogallonic acid
P9	3.17	633.0677	−8.9	C_27_H_22_O_18_	633 (100), 463 (6), 301 (66), 275 (7)	Corilagin
P10	3.31	1235.0712	0.8	C_55_H_32_O_34_	1235 (19), 617 (100)	*O*-Galloyl punicalagin
P11	3.45	163.0401	0.2	C_9_H_8_O_3_	163 (3), 119 (100), 117 (8), 93 (62)	*p*-Coumaric acid (**6**)
		600.9896	−0.01	C_28_H_10_O_16_	601 (100), 583 (3), 301 (18), 298 (20), 271 (22), 243 (5), 214 (2)	Terminalin
P12	3.84	433.0397	−3.6	C_19_H_14_O_12_	433 (78), 301 (77), 300 (100), 272 (4), 244 (7), 216 (10), 200 (5), 172 (6), 132 (4)	Ellagic acid pentoside
P13	3.93	953.0899	−0.3	C_41_H_30_O_27_	953 (100), 463 (2), 301 (61), 275 (5), 205 (4), 169 (2)	Chebulagic acid (**18**)
P14	4.02	300.9978	−4.0	C_14_H_6_O_8_	301 (100), 284 (7), 257 (2), 245 (3), 229 (4), 201 (3), 185 (3), 173 (3), 161 (1), 145 (5)	Ellagic acid (**12**)
P15	4.20	431.0969	−3.4	C_21_H_20_O_10_	431 (48), 341 (25), 323 (6), 311 (100), 283 (54), 269 (6), 161 (6), 117 (8)	Flavon-*C*-glucoside (Vitexin)
P16	4.42	955.1002	−5.9	C_41_H_32_O_27_	955 (100), 937 (6), 785 (4), 617 (3), 465 (4), 337 (3), 319 (4), 275 (8), 231 (11), 205 (6), 169 (4)	Chebulinic acid
P17	4.53	477.1006	−6.8	C_22_H_22_O_12_	477 (100), 313 (6), 265 (26), 235 (7), 211 (5), 205 (7), 169 (37), 163 (6)	Galloyl-coumaroyl-glucose (**1**)
P18	4.95	315.0126	−6.5	C_15_H_8_O_8_	315 (11), 300 (100), 271 (7), 216 (12), 200 (6), 160 (7), 132 (7)	*O*-Methyl ellagic acid
		583.1075	−3.1	C_28_H_24_O_14_	583 (100), 431 (22), 341 (31), 323 (8), 311 (75), 283 (40), 271 (14), 241 (10), 211 (9), 169 (18), 125 (8)	Galloylvitexin isomer I
P19	5.42	583.1085	−1.4	C_28_H_24_O_14_	583 (84), 431 (37), 341 (55), 311 (100), 323 (17), 283 (53), 271 (27), 211 (14), 169 (23), 125 (9)	Galloylvitexin isomer II
P20	5.79	735.1184	−2.6	C_35_H_28_O_18_	735 (100), 583 (90), 565 (45), 431 (24), 341 (25), 311 (39), 293 (42), 271 (24), 211 (20), 169 (38), 125 (8)	Digalloylvitexin isomer
		545.2010	-3.4	C_28_H_34_O_11_	545 (100), 307 (6), 265 (37), 235 (15), 219 (18), 205 (34), 201 (9), 177 (11), 163 (43), 145 (69), 119 (16)	e.g., Cinnamrutinose B
P21	6.24	329.0293	−3.0	C_16_H_10_O_8_	329 (8), 314 (55), 298 (85), 271 (100), 243 (48), 214 (29), 187 (21), 159 (24), 131 (12), 103 (7), 75 (6)	Di-*O*-methyl ellagic acid
P22	7.80	343.0443	−4.8	C_17_H_12_O_8_	343 (9), 328 (45), 313 (100), 297 (87), 285 (22), 269 (77), 241 (16), 213 (30), 185 (32), 157 (19), 130 (12)	Tri-*O*-methyl ellagic acid

* Peak numbers correspond to Figure 1.

**Table 2:** NMR data (Methanol-*d*_4_, 600/150 MHz) of compound **1**.

Moiety	No.	δ_C_, Type	δ_H_ (*Multiplicity*, *J*)	HMBC	COSY
Glucose	1*α*	95.9, CH	5.66 (*d*, 3.3, 0.5 H)	3, 4, 7′	2
	1*β*		5.66 (*d*, 7.7, 0.5 H)	3, 4, 7′	2
	2	74.1, CH	3.46–3.52 (*m*, 2 H)	1, 3, 4	1, 3
	3	78.1, CH	3.46–3.52 (*m*, 2 H)	2, 3, 4	2, 4
	4	71.3, CH	3.41–3.46 (*m*, 1 H)	3, 5	3, 5
	5	76.3, CH	3.65–3.70 (*m*, 1 H)	1, 3, 4	4, 6a, 6b
	6a	64.4, CH_2_	4.50 (*dd*, 12.2, 2.8 Hz, 1 H)	4, 5, 9″	5, 6b
	6b		4.31 (*dd*, 12.2, 5.6 Hz, 1 H)	4, 5, 9″	5, 6a
Galloyl	1′	120.5, C	-	-	-
	2′/6′	110.5, CH	7.13 (*s*, 2 H)	1′, 2′, 3′, 4′, 5′, 6′, 7′	-
	3′/5′	146.6, C	-		-
	4′	140.7, C	-		-
	7′	166.9, C	-		-
Coumaroyl	1″	127.2, C	-	-	-
	2″/6″	131.2, CH	7.45 (*d*, 8.5 Hz, 2 H)	2″, 4″, 6″, 7″	3″, 5″
	3″/5″	116.9, CH	6.80 (*d*, 8.5 Hz, 2 H)	1″, 3″, 4″, 5″	2″, 6″
	4″	161.3, C	-	-	-
	7″	146.9, CH	7.63 (*d*, 15.7 Hz, 1 H)	1″, 2″, 6″, 8″, 9″	8″
	8″	114.9, CH	6.36 (*d*, 15.7 Hz, 1 H)	1′, 9′	7″
	9″	169.1, C	-	-	-

**Table 3:** Antifungal (*Septoria tritici*, *Botrytis cinerea*), antioomycotic (*Phytophthora infestans*), and antibacterial (*Aliivibrio fisheri*) activities of crude extract and isolated compounds from *T*. *dhofarica*.

	Growth Inhibition [%] *			
	Antifungal Assays			Antibacterial Assay
	*S. tritici*	*B. cinerea*	*P. infestans*	*A. fischeri*
**Crude extract**	**100 µg/mL**	**100 µg/mL**	**100 µg/mL**	**500 µg/mL**
	79 ± 2	29 ± 9	82 ± 5	100 ± 1
**Compound**	**100 µM**	**100 µM**	**100 µM**	**100 µM**
**1**	46 ± 1	1 ± 6	79 ± 7	−28 ± 6
**2**	41 ± 1	−13 ± 7	61 ± 10	−51 ± 10
**3**	4 ± 5	11 ± 3	13 ± 2	−29 ± 3
**4**	34 ± 3	−22 ± 15	31 ± 1	−5 ± 3
**5**	−21 ± 14	−25 ± 14	6 ± 1	−31 ± 5
**6**	92 ± 1	−11 ± 28	10 ± 5	−6 ± 5
**7**	35 ± 4	−4 ± 8	30 ± 8	−114 ± 8
**8**	44 ± 1	12 ± 2	73 ± 4	−76± 11
**9**	51 ± 1	6 ± 8	81 ± 1	−77 ± 7
**10**	54 ± 3	14 ± 3	79 ± 1	−51 ± 3
**11**	14 ± 1	15 ± 8	32 ± 11	−22 ± 5
**12**	−2 ± 8	23 ± 4	63 ± 4	−389 ± 26
**13**	19 ± 7	4 ± 7	93 ± 1	−384 ± 27
**14**	36 ± 1	−6 ± 7	2 ± 4	−17 ± 7
**15**	38 ± 3	−35 ± 34	39 ± 4	−172 ± 11
**16**	−119 ± 26	20 ± 2	0 ± 2	−17 ± 5
**17**	46 ± 2	−3 ± 4	82 ± 1	−350 ± 111
**18**	13 ± 4	2 ± 4	19 ± 2	−23 ± 4
**19**	61 ± 3	−17 ± 5	94 ± 1	−643 ± 10
**20**	−4 ± 5	8 ± 4	94 ± 1	−564 ± 45

* Negative values indicate an increase in fungal or bacterial growth in comparison to the negative control (0% inhibition). Data represent mean values ± standard deviation (n = 6 for antibacterial assay, n = 3 for antifungal assays).

## Data Availability

The original contributions presented in the study are included in the article and the Supplementary Material. The raw data supporting the conclusions of this article will be made available by the corresponding authors upon request. All primary data and reference compounds are stored at the IPB primary data storage for 10+ years and in the compound depository to the extent available or stable.

## Supplementary Materials

The following supporting information can be downloaded at https://www.mdpi.com/article/10.3390/molecules30040952/s1, Figure S1: Antibacterial assay against Gram-negative *A. fischeri*; Figure S2: Antifungal assay against *P. infestans*; Figure S3: Antifungal assay against *S. tritici*; Figure S4: Antifungal assay against *B. cinerea*; Figure S5: TWC and TIC obtained by UHPLC-ESI-HRMS of the crude extract from *T. dhofarica*; Figure S6: 1H spectrum of compound **1**; Figure S7: 13C spectrum of compound **1**; Figure S8: COSY spectrum of compound **1**; Figure S9: HSQC spectrum of compound **1**; Figure S10: HMBC spectrum of compound **1**; Full spectroscopic data set of compounds **1**–**20**.
