# Supplementary material for: Profiling and Bioactivity of Polyphenols from the Omani Medicinal Plant Terminalia dhofarica (syn. Anogeissus dhofarica)
Source: Molecules. 2025 Feb 18;30(4):952. doi: 10.3390/molecules30040952 (PMC11858248; doi:10.3390/molecules30040952)
Supplement: Supplementary file 1 [file molecules-30-00952-s001.zip › molecules-3444806-supplementary.pdf]

Supporting Information

Profiling and Bioactivity of Polyphenols from the Omani medicinal plant *Terminalia dhofarica* (syn. *Anogeissus dhofarica*)

Jonas Kappen[1], Luay Rashan[2], Katrin Franke[1]*, Ludger A. Wessjohann[1,3]*

[1] Department of Bioorganic Chemistry, Leibniz Institute of Plant Biochemistry (IPB), 06120 Halle (Saale), Germany
[2] Biodiversity Unit, Research Center, Dhofar University, Salalah, Oman.
[3] Institute of Chemistry, Martin Luther University Halle-Wittenberg, 06120 Halle (Saale), Germany

* Correspondence: Katrin Franke, kfranke@ipb-halle.de, Tel: +49-345-5582-1380 (K.F.)
Ludger Wessjohann, wessjohann@ipb-halle.de, Tel: +49-345-5582-1301 (L.A.W.)

Content **page**

Figure S1 – S4. Screening for antibacterial and antifungal biological activities 2

Figure S5. TWC and TIC obtained by UHPLC-ESI-HRMS of the crude extract from *T. dhofarica* 4 5

Figure S6 – S10. 1D and 2D NMR spectra of compound **1** 8

Full spectroscopic data set of compounds **1-20**

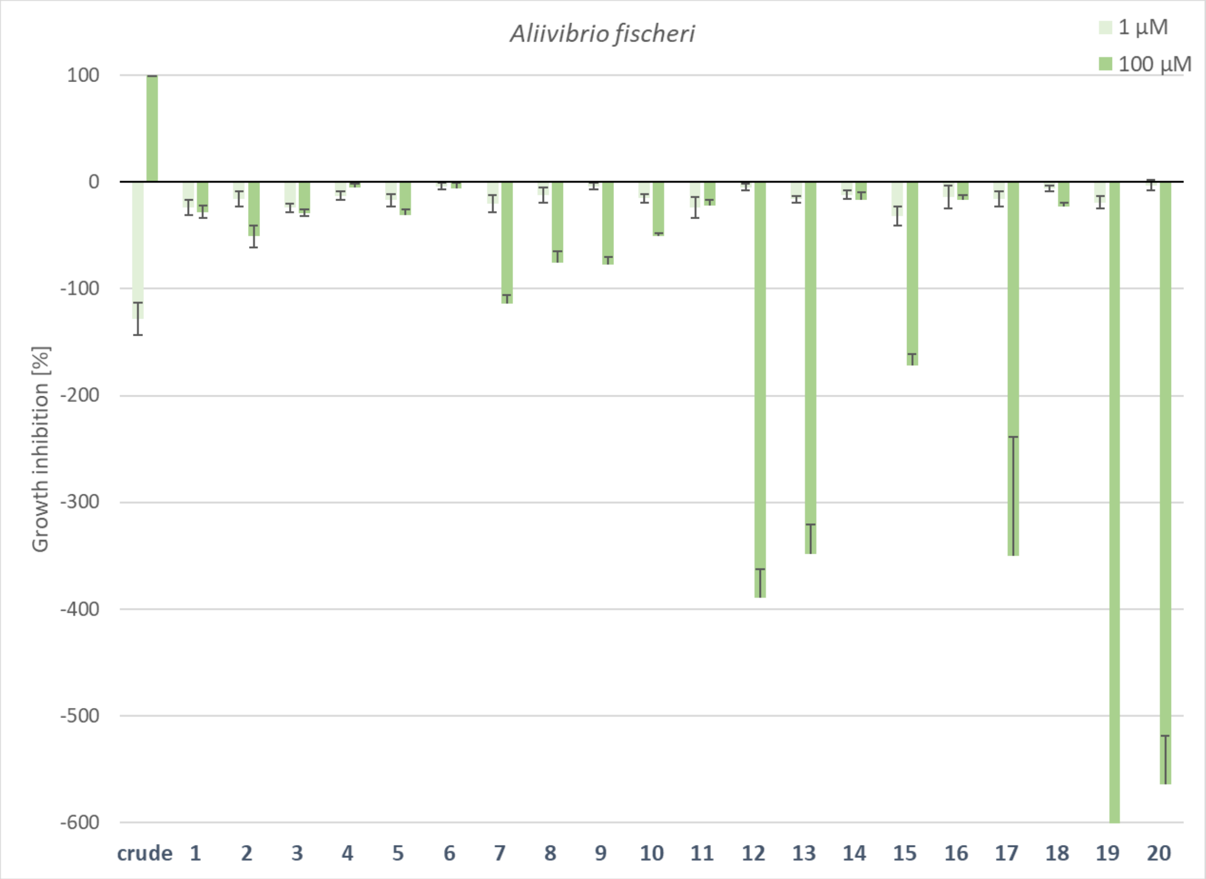

Figure S1. Antibacterial activity of *T. dhofarica* crude extract (50 and 500 µg/mL) and isolated compounds **1-20** against Gram-negative *A. fischeri*.

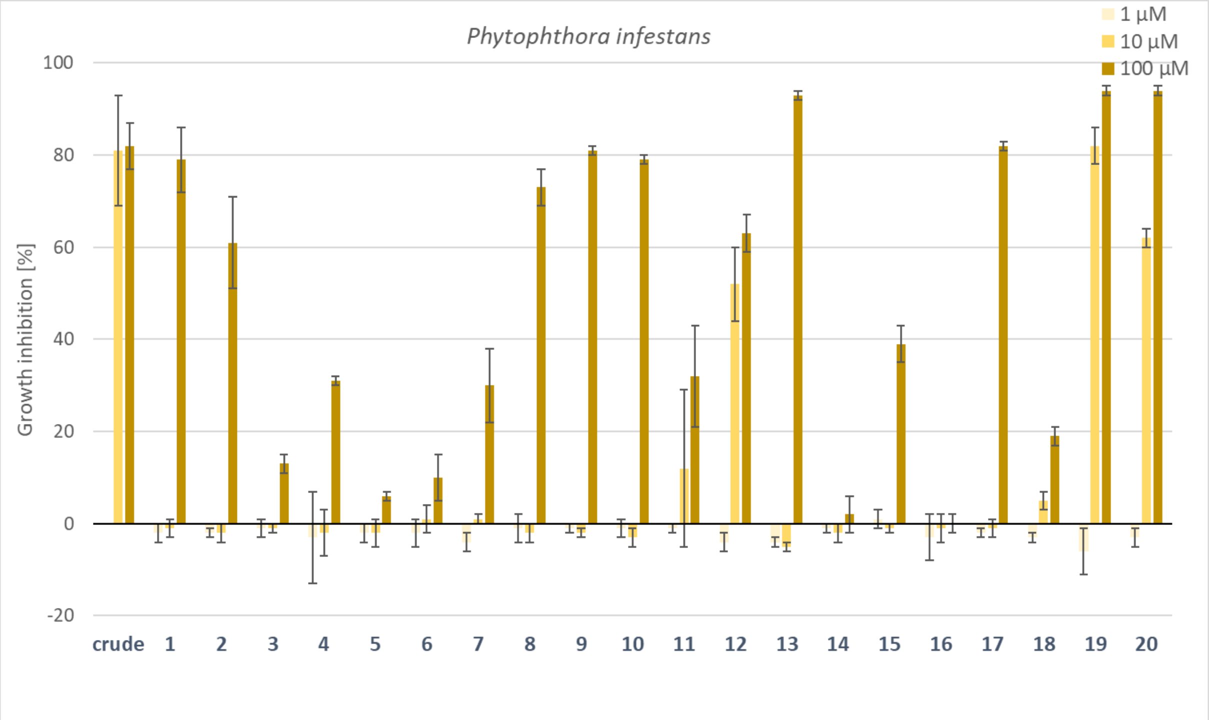

Figure S2. Antifungal activity of *T. dhofarica* crude extract (10 and 100 µg/mL) and isolated compounds **1-20** against the oomycete *P. infestans*.

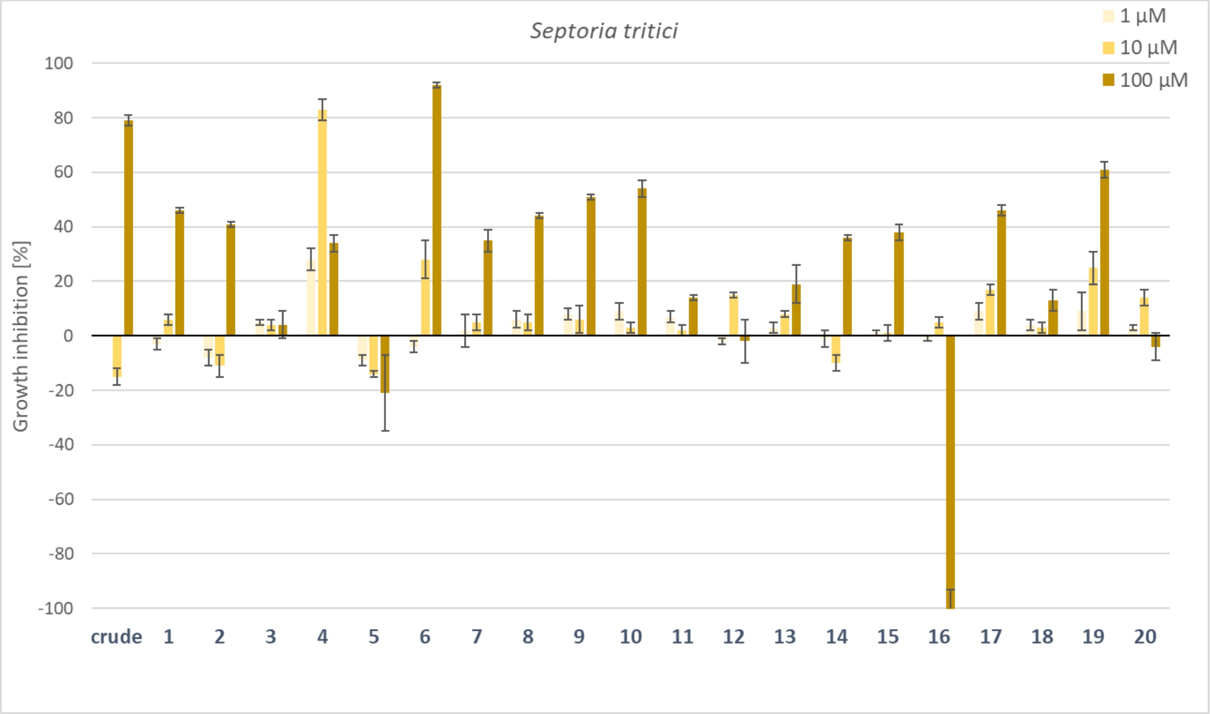

Figure S3. Antifungal activity of *T. dhofarica* crude extract (10 and 100 µg/mL) and isolated compounds **1-20** against *S. tritici*.

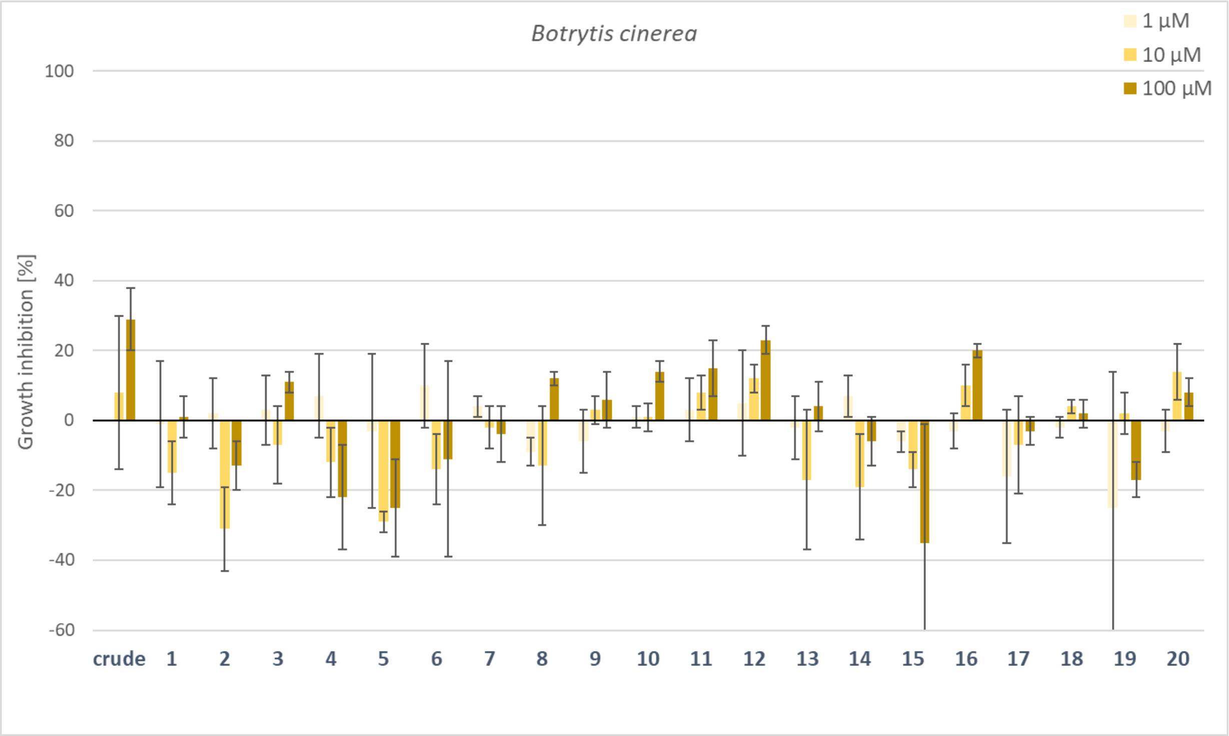

Figure S4. Antifungal activity of *T. dhofarica* crude extract (10 and 100 µg/mL) and isolated compounds **1-20** against *B. cinerea*.

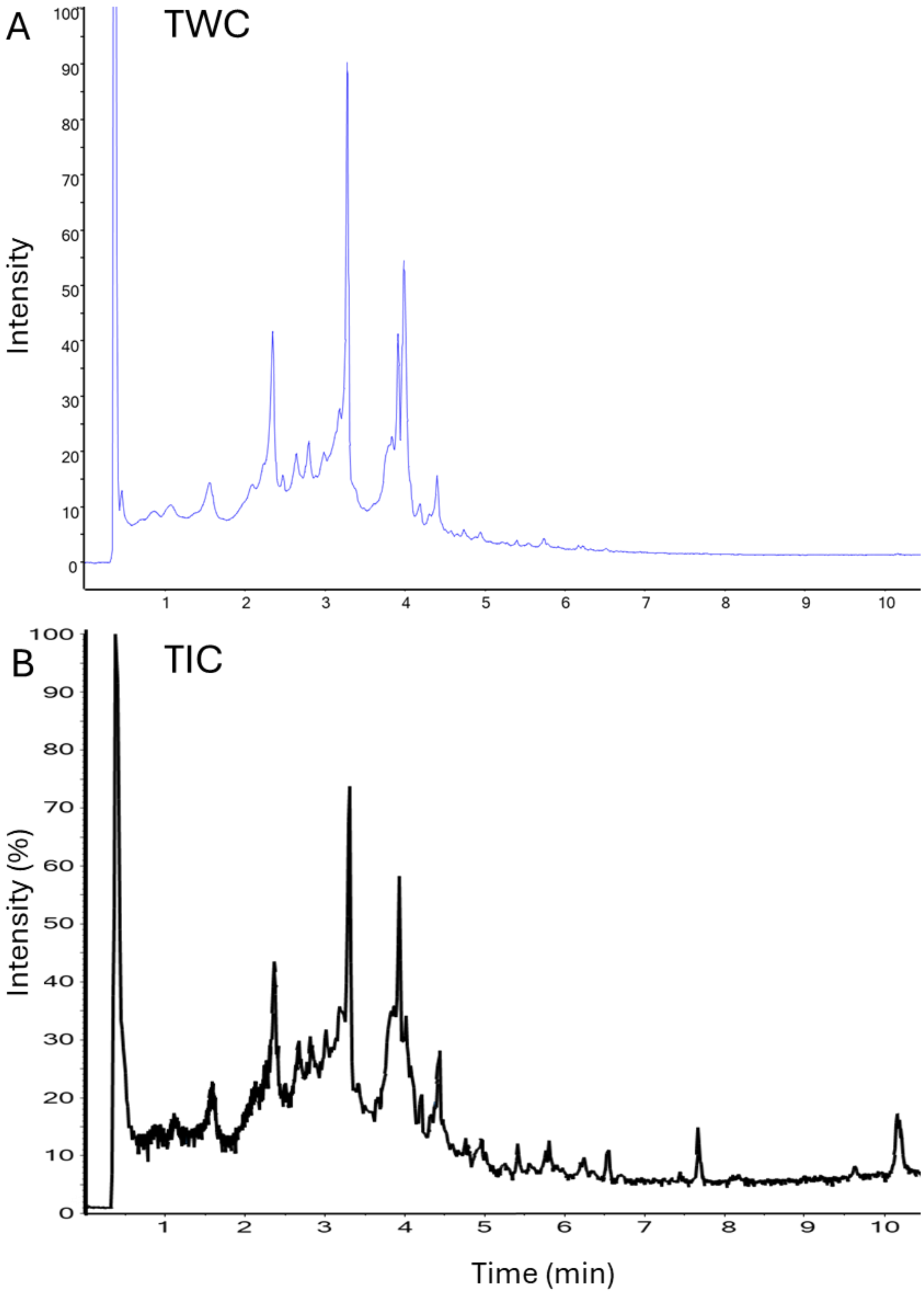

Figure S5. A) Corresponding total wavelength chromatogram (TWC) and **B)** Total ion chromatogram (TIC) obtained by UHPLC-ESI-HRMS of the crude extract from *T. dhofarica*

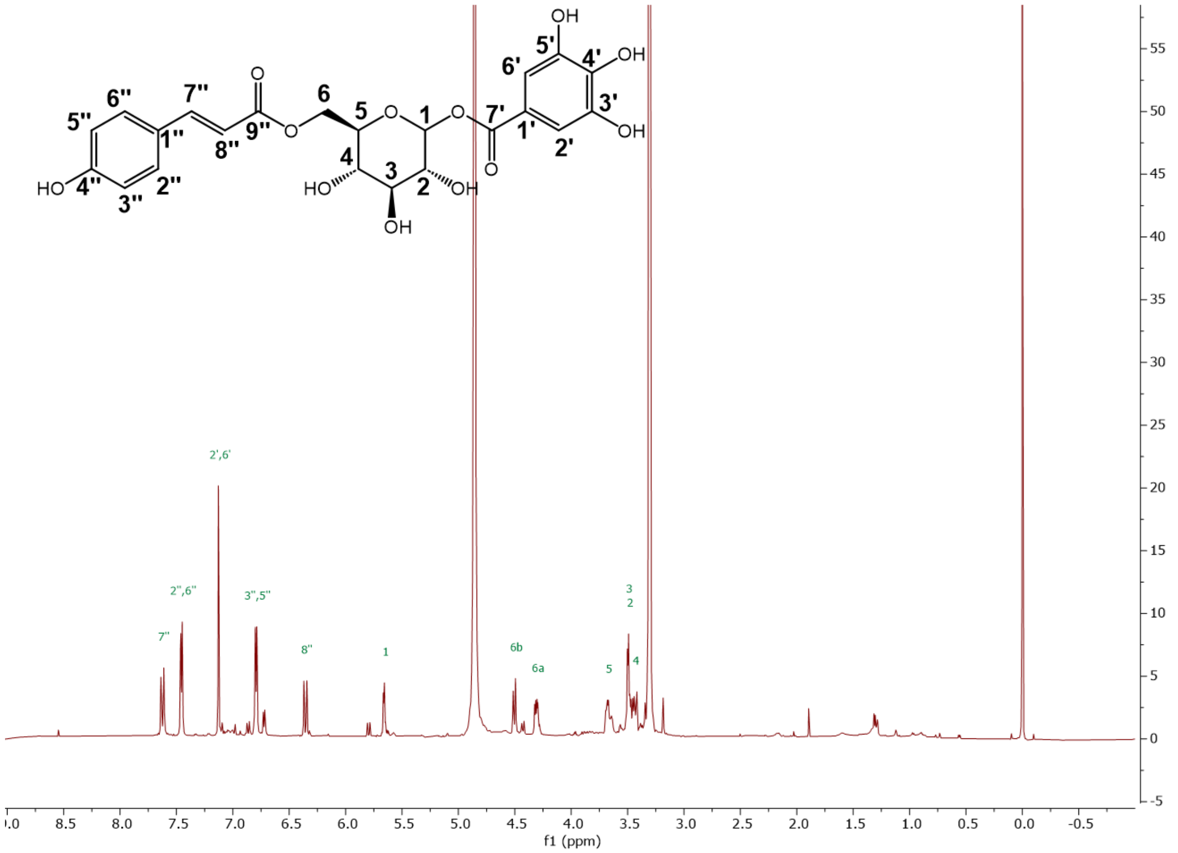

Figure S6. ^{1}H NMR spectrum of compound **1**, MeOD, 25°C, 600 MHz, 40 scans

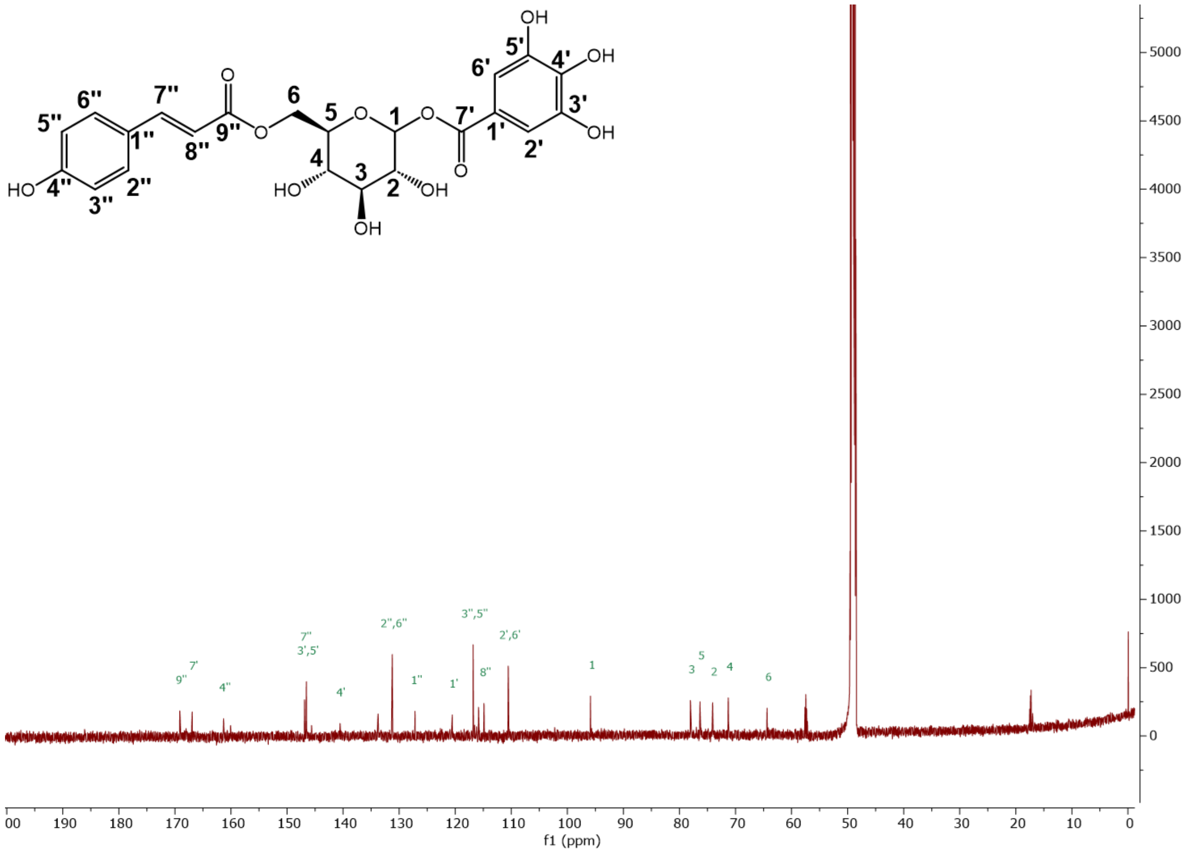

Figure S7. ^{13}C NMR spectrum of compound **1**, MeOD, 25°C, 150 MHz, 25,000 scans

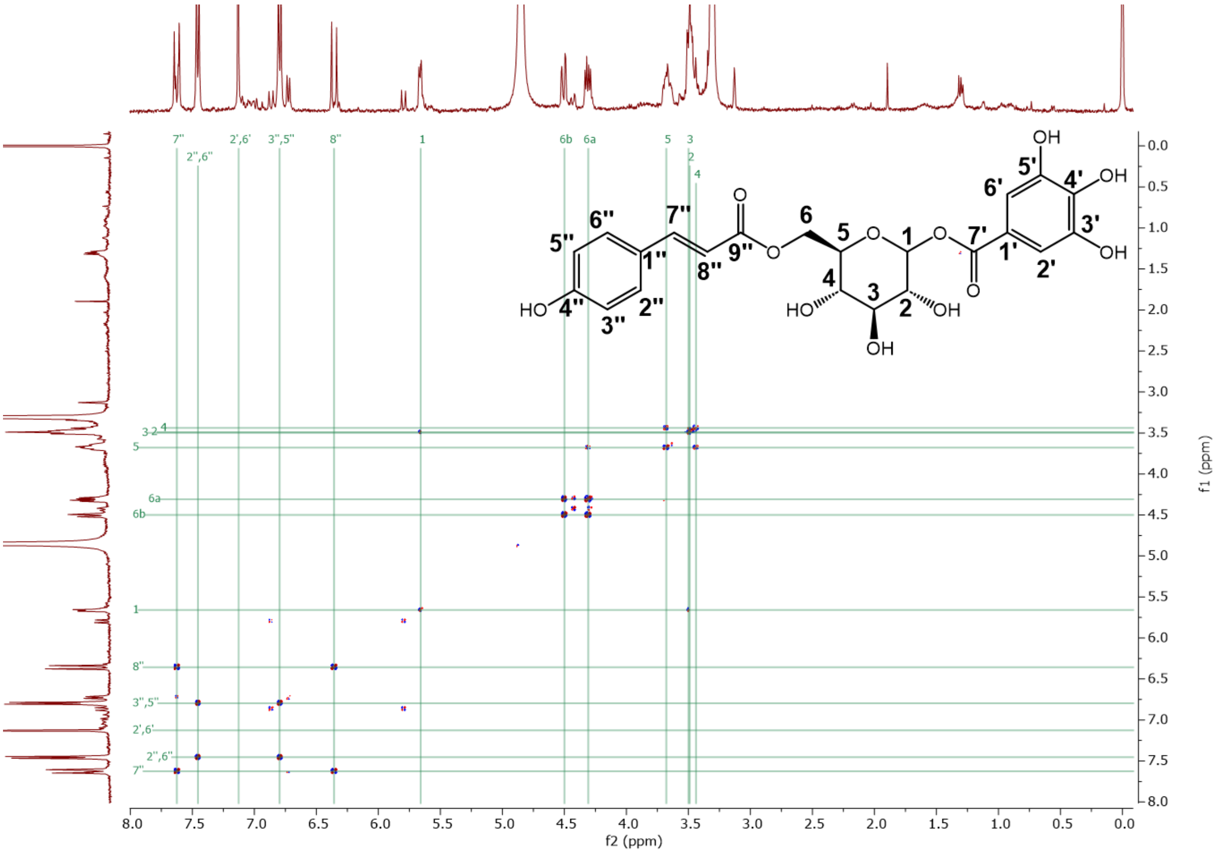

Figure S8. COSY spectrum of compound **1**, MeOD, 25°C, 600 MHz/600 MHz, 25 scans

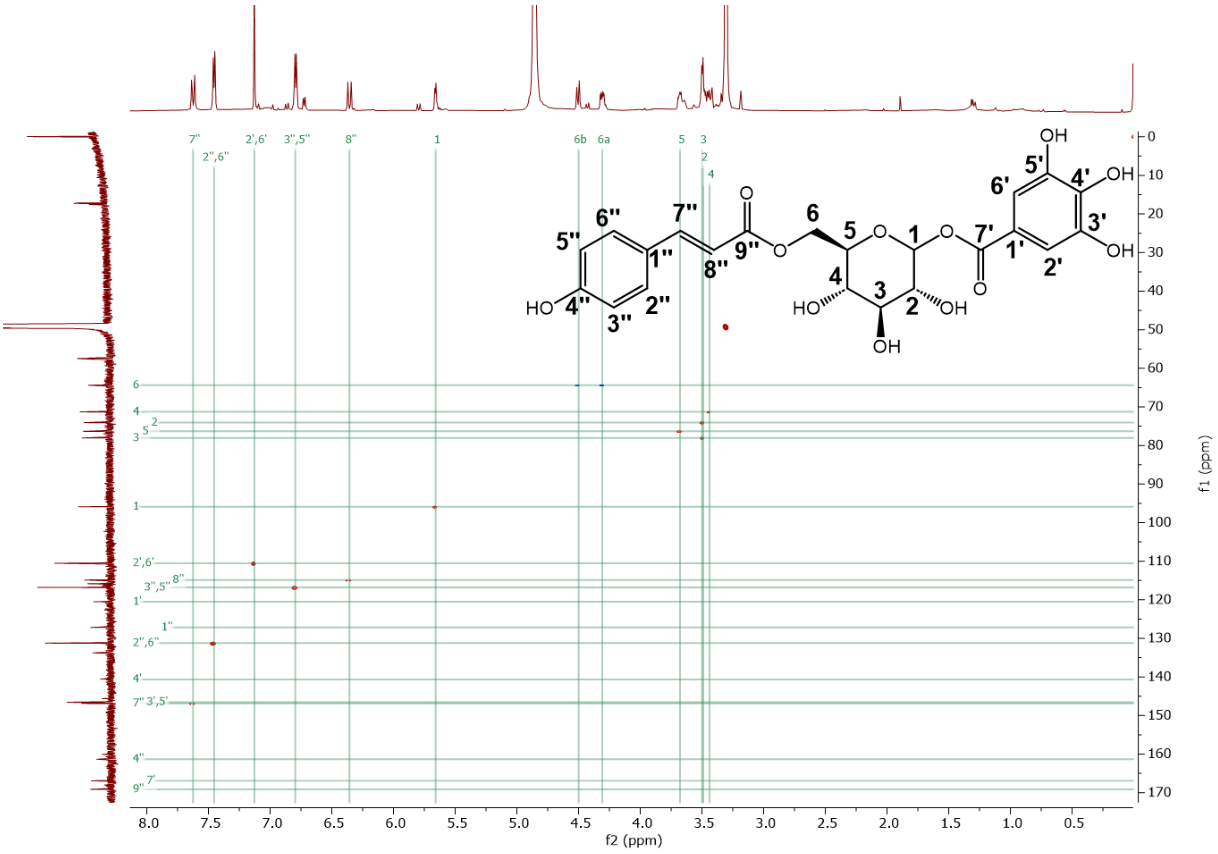

Figure S9. HSQC spectrum of compound **1**, MeOD, 25°C, 600 MHz/150 MHz, 32 scans

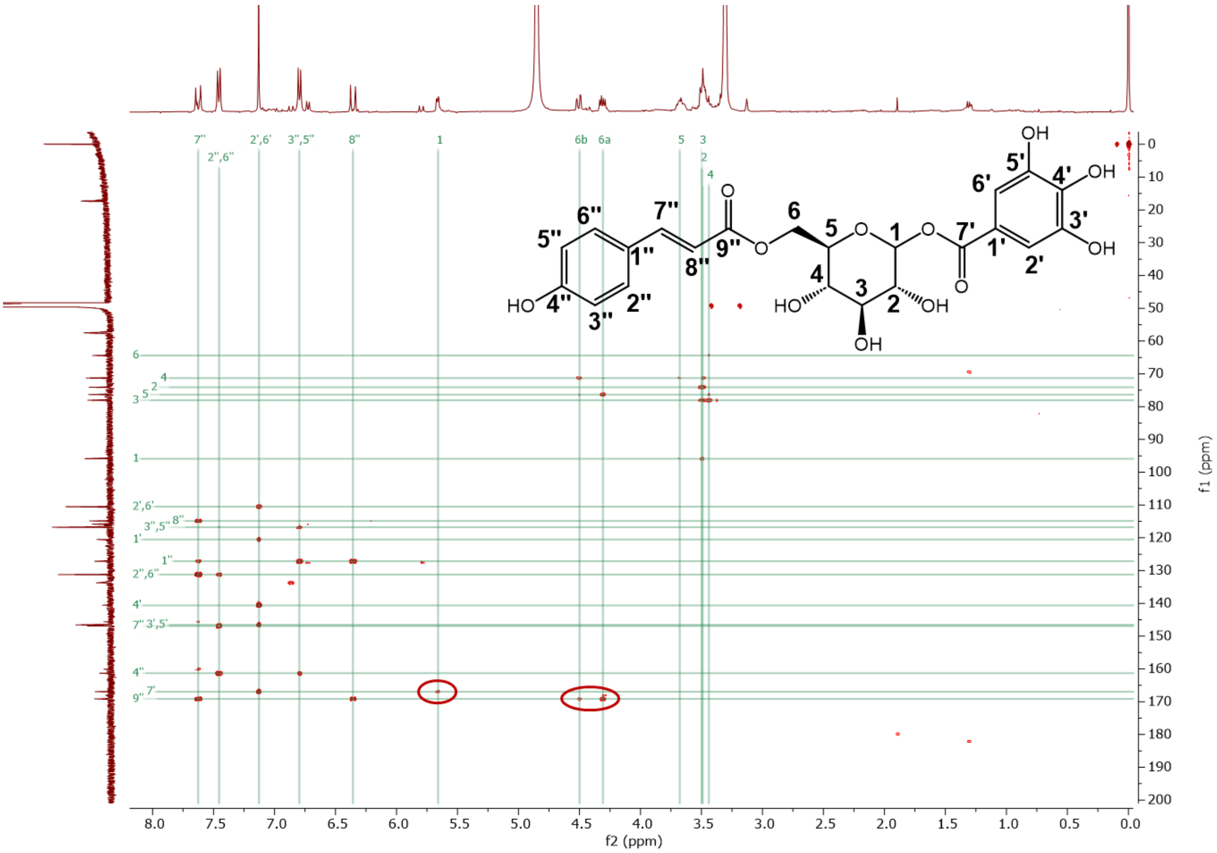

Figure S10. HMBC spectrum of compound **1**, MeOD, 25°C, 600 MHz/150 MHz, 80 scans. Two red rings mark the crucial correlations for the connection pattern of H-1 and H-6a/b.

Full spectroscopic data set of compounds 1-20

1-*O*-Galloyl-6-*O*-*trans*-*p*-coumaroyl-D-glucopyranose (**1**): white solid; ^{1}H NMR (600 MHz, Methanol-*d*$_4$) δ 7.63 (1H, *d*, *J* = 15.7 Hz, H-7''), 7.45 (2H, *d*, *J* = 8.5, H-2'' + H-6''), 7.13 (2H, *s*, H-2' + H-6'), 6.80 (2H, *d*, *J* = 8.5 Hz, H-3'' + H-5''), 6.36 (1H, *d*, *J* = 15.7 Hz, H-8''), 5.66 (1H, *d*, *J* = 7.7, H-1*β*), 5.66 (1H, *d*, *J* = 3.3, H-1*β*), 4.50 (1H, *d*, *J* = 12.0Hz, H-6a), 4.31 (1H, *dd*, *J* = 12.0, 5.6 Hz, H-6b), 3.68 (1H, *m*, H-5), 3.51-3.40 (3H, *m*, H-2 + H-3 + H-4); ^{13}C NMR (150 MHz, Methanol-*d*$_4$) δ 169.1 (C, C-9''), 166.9 (C, C-7'), 161.3 (C, C-4''), 146.9 (CH, C-7''), 146.6 (2C, C-3' + C-5'), 140.7 (C, C-4'), 131.2 (2CH, C-2'' + C-6''), 127.2 (C, C-1''), 120.5 (C, C-1'), 116.8 (2CH, C-3'' + C-5''), 114.9 (CH, C-8''), 110.5 (2CH, C-2' + C-6'), 95.9 (CH, C-1), 78.1 (CH, C-3), 76.3 (CH, C-5), 74.1 (CH, C-2), 71.3 (CH, C-4), 64.4 (CH_2, C-6); HR-ESI-MS (TOF) *m/z* $[M-H]^-$ 477.1030 (calc for $C_{22}H_{22}O_{12}^-$, 477.1033); MS^2-fragmentation (CE = −40 V) *m/z* 477 (5), 313 (5), 265 (45), 235 (10), 211 (13), 205 (48), 177 (7), 169 (100), 163 (53), 161 (16), 151 (20), 145 (21), 125 (32), 123 (27), 119 (17). MS-data corresponds to literature [16].

p-Hydroxybenzaldehyde (**2**): pale yellow solid; ^{1}H NMR (500 MHz, DMSO-*d*$_6$) δ 9.79 (1H, s, H-7), 7.76 (2H, *d*, *J* = 8.3 Hz, H-2 + H-6), 6.93 (2H, *d*, *J* = 8.3 Hz, H-3 + H-5); ^{13}C NMR (obtained from HSQC and HMBC, DMSO-*d*$_6$) δ 190.6 (CH, C-7), 163.4 (C, C-4), 132.1 (2CH, C-2 + C-6), 128.4 (C, C-1), 115.9 (2CH, C-3 + C-5); HR-ESI-MS (TOF) *m/z* $[M-H]^-$ 121.0287 (calc for $C_7H_5O_2^-$, 121.0290); MS^2-fragmentation (CE = −20 V) *m/z* 121 (86), 92 (100), 65 (3). Data corresponds to literature [17,18].

Protocatechuic acid (**3**): white amorphous powder; ^{1}H NMR (600 MHz, DMSO-*d*$_6$) δ 12.13 (1H, *brs*, 7-COOH), 9.34 (2H, *brs*, 3-OH + 4-OH), 7.33 (1H, *d*, *J* = 2.1 Hz, H-2), 7.28 (1H, *dd*, *J* = 8.2, 2.1 Hz, H-6), 6.78 (1H, *d*, *J* = 8.2 Hz, H-5); 13C NMR (obtained from HSQC and HMBC, DMSO-*d*$_6$) δ 167.4 (C, C-7), 149.8 (C, C-4), 144.6 (C, C-3), 121.7 (C, C-6), 121.4 (CH, C-1), 116.4 (CH, C-2), 115.0 (CH, C-5); HR-ESI-MS (TOF) *m/z* $[M-H]^-$ 153.0193 (calc for $C_7H_5O_4^-$, 153.0188); MS^2-fragmentation (CE = −30 V) *m/z* 153 (3), 109 (100), 108 (56), 91 (10), 81 (8), 65 (9). Data corresponds to literature [19].

Gallic acid (**4**): white solid; ^{1}H NMR (400 MHz, DMSO-*d*$_6$) δ 6.86 (2H, *s*, H-2 + H-6); ^{13}C NMR (100 MHz, DMSO-*d*$_6$) δ 170.3 (C, C-7), 145.5 (2C, C-3 + C-5), 137.0 (C, C-4), 125.8 (C, C-1), 108.9 (2CH, C-2 + C-6); HR-ESI-MS (TOF) *m/z* $[M-H]^-$ 169.0152 (calc for $C_7H_5O_5^-$, 169.0137); MS^2-fragmentation (CE = −50 V) *m/z* 125 (50), 124 (77), 107 (7), 97 (10), 81 (11), 79 (100), 69 (39), 67 (30), 53 (12), 51 (98), 45 (7), 43 (9), 41 (39). Data corresponds to literature [20].

7-*O*-Methyl gallate (**5**): white amorphous solid; ^{1}H NMR (400 MHz, Methanol-*d*$_4$) δ 7.03 (2H, *s*, H-2 + H-6), 3.81 (3H, *s*, 7-OMe); ^{13}C NMR (obtained from HSQC and HMBC, Methanol-*d*$_4$) δ 169.2 (C, C-7), 146.7 (2C, C-3 + C-5), 140.1 (C, C-4), 121.0 (C, C-1), 110.1 (2CH, C-2 + C-6), 52.2 (CH_3, 7-OMe); HR-ESI-MS (TOF) *m/z* $[M-H]^-$ 183.0296 (calc for $C_8H_7O_5^-$, 183.0293); MS^2-fragmentation (CE = −20 V) *m/z* 183 (100), 168 (13), 124 (88). Data corresponds to literature [21,22].

trans-*p*-Coumaric acid (**6**): white solid; ^{1}H NMR (500 MHz, Methanol-*d*$_4$) δ 7.59 (1H, *d*, *J* = 15.9 Hz, H-7), 7.45 (2H, *d*, *J* = 8.6 Hz, H-2 + H-6), 6.80 (2H, *d*, *J* = 8.6 Hz, H-3 + H-5), 6.28 (1H, *d*, *J* = 15.9 Hz, H-8); ^{13}C NMR (126 MHz, Methanol-*d*$_4$) δ 171.1 (CH, C-9), 161.1 (C, C-4), 146.5 (CH, C-7), 131.1 (2CH, C-2 + C-6), 127.3 (C, C-1), 116.8 (2CH, C-3 + C-5), 115.8 (C, C-8); HR-ESI-MS (TOF) *m/z* $[M-H]^-$ 163.0393 (calc for $C_9H_7O_3^-$, 163.0395); MS^2-fragmentation (CE = −45 V) *m/z* 119 (100), 117 (12), 93 (35), 91 (4), 65 (3). Data corresponds to literature [23].

Chebulic acid (**7**): yellow solid; ^{1}H NMR (400 MHz, Methanol-*d₄*) δ 7.04 (1H, *s*, H-8), 5.23 (1H, *s*, H-3), 3.86 (1H, *d*, *J* = 8.6 Hz, H-4), 3.11 (1H, *td*, *J* = 9.4, 4.4 Hz, H-9), 2.87 (1H, *dd*, *J* = 17.1, 10.4 Hz, H-10a), 2.35 (1H, *dd*, *J* = 17.1, 4.4 Hz, H-10b); ^{13}C NMR (100 MHz, Methanol-d_4) δ 176.9 (C, C-13), 175.5 (C, C-11), 172.6 (C, C-12), 166.8 (C, C-1), 146.7 (C, C-7), 144.0 (C, C-5), 140.6 (C, C-6), 118.1 (C, C-4a), 116.3 (C, C-8a), 109.3 (CH, C-8), 79.0 (CH, C-3), 45.5 (CH, C-9), 37.4 (CH, C-4), 35.2 (CH_2, C-10). HR-ESI-MS (TOF) *m/z* $[M-H]^-$ 355.0302 (calc for $C_{14}H_{12}O_{11}^-$, 355.0301); MS^2-fragmentation (CE = − 45 V) *m/z* 355 (30), 337 (100), 249 (28), 205 (43), 193 (37), 187 (5), 179 (12), 163 (22), 161 (14), 149 (10), 135 (5). Data corresponds to literature [24].

12-*O*-Methyl chebulic acid (**8**): yellow solid; ^{1}H NMR (600 MHz, Methanol-d_4) δ 7.04 (1H, *s*, H-8), 5.34 (1H, *s*, H-3), 3.84 (1H, *d*, *J* = 9.1 Hz, H-4), 3.62 (3H, *s*, H-12OMe), 3.12 (1H, *td*, *J* = 9.6, 4.4 Hz, H-9), 2.87 (1H, *dd*, *J* = 17.1, 10.4 Hz, H-10a), 2.35 (1H, *dd*, *J* = 17.1, 4.4 Hz, H-10b); ^{13}C NMR (150 MHz, Methanol-d_4) δ 176.7 (C, C-13), 175.3 (C, C-11), 171.3 (C, C-12), 166.3 (C, C-1), 146.8 (C, C-7), 144.0 (C, C-5), 140.7 (C, C-6), 117.7 (C, C-4a), 115.9 (C, C-8a), 109.3 (CH, C-8), 78.7 (CH, C-3), 53.3 (CH_3, 12-OMe), 45.2 (CH, C-9), 37.2 (CH, C-4), 35.1 (CH_2, C-10); HR-ESI-MS (TOF) *m/z* $[M-H]^-$ 369.0554 (calc for $C_{15}H_{13}O_{11}^-$, 369.0458); MS^2-fragmentation (CE = −45 V) *m/z* 369 (3), 351 (83), 307 (12), 251 (49), 231 (100), 219 (7), 207 (25), 205 (18), 203 (83), 192 (11), 187 (16), 177 (19), 175 (29), 163 (11), 159 (15), 147 (13), 135 (10). Data corresponds to literature [25].

11,12-*O*-Dimethyl chebulic acid (**9**): yellow solid; ^{1}H NMR (600 MHz, Methanol-d_4) δ 7.03 (1H, *s*, H-8), 5.34 (1H, *s*, H-3), 3.88 (1H, *d*, *J* = 9.1 Hz, H-4), 3.62 (3H, *s*, 12-OMe), 3.51 (3H, *s*, 11-OMe), 3.17 (1H, *td*, *J* = 9.1, 5.6 Hz, H-9), 2.81 (1H, *dd*, *J* = 17.1, 8.8 Hz, H-10a), 2.46 (1H, *dd*, *J* = 17.1, 5.6 Hz, H-10b); ^{13}C NMR (150 MHz, Methanol-d_4) δ 176.6 (C, C-13), 173.8 (C, C-11), 171.3 (C, C-12), 166.3 (C, C-1), 146.9 (C, C-7), 144.1 (C, C-5), 140.8 (C, C-6), 117.5 (C, C-4a), 116.2 (C, C-8a), 109.3 (CH, C-8), 78.9 (CH, C-3), 53.3 (CH_3, 12-OMe), 52.2 (CH_3, 11-OMe), 45.1 (CH, C-9), 37.1 (CH, C-4), 35.1 (CH_2, C-10); HR-ESI-MS (TOF) *m/z* $[M-H]^-$ 383.0613 (calc for $C_{16}H_{15}O_{11}^-$, 383.0614); MS^2-fragmentation (CE = −45 V) *m/z* 351 (100), 307 (6), 251 (29), 231 (63), 207 (12), 203 (48), 187 (7), 177 (9), 175 (12), 159 (6). Data corresponds to literature [25,26].

12,13-*O*-Dimethyl chebulic acid (**10**): yellow solid; ^{1}H NMR (600 MHz, Methanol-d_4) δ 7.03 (1H, *s*, H-8), 5.27 (1H, *d*, *J* = 1.1 Hz, H-3), 3.88 (1H, *d*, *J* = 7.2, 1.1 Hz, H-4), 3.67 (3H, *s*, 13-OMe), 3.63 (3H, *s*, 12-OMe), 3.17 (1H, *td*, *J* = 9.1, 5.6 Hz, H-9), 2.81 (1H, *dd*, *J* = 17.1, 8.8 Hz, H-10a), 2.46 (1H, *dd*, *J* = 17.1, 5.6 Hz, H-10b); ^{13}C NMR (150 MHz, Methanol-d_4) δ 175.22 (C, C-13), 175.18 (C, C-11), 171.3 (C, C-12), 166.2 (C, C-1), 146.9 (C, C-7), 143.9 (C, C-5), 140.9 (C, C-6), 117.6 (C, C-4a), 116.1 (C, C-8a), 109.1 (CH, C-8), 78.9 (CH, C-3), 53.4 (CH_3, 12-OMe), 52.8 (CH_3, 13-OMe), 45.3 (CH, C-9), 37.4 (CH, C-4), 35.0 (CH_2, C-10); HR-ESI-MS (TOF) *m/z* $[M-H]^-$ 383.0610 (calc for $C_{16}H_{15}O_{11}^-$, 383.0614); MS^2-fragmentation (CE = −45 V) *m/z* 351 (90), 307 (7), 263 (5), 251 (46), 231 (100), 207 (20), 203 (80), 192 (8), 187 (12), 177 (20), 175 (22), 163 (8), 159 (10), 147 (9). Data corresponds to literature [25,26].

11-*O*-Methyl brevifolincarboxylate (**11**): white amorphous solid; ^{1}H NMR (500 MHz, Methanol-*d*$_4$) δ 7.35 (1H, *s*, H-7), 4.55 (1H, *dd*, *J* = 7.7, 2.2 Hz, H-8), 3.73 (3H, *s*, 11-OMe), 3.01 (1H, *dd*, *J* = 18.9, 7.7 Hz, H-9a), 2.51 (1H, *dd*, *J* = 18.9, 2.2 Hz, H-9b); ^{13}C NMR (obtained from HSQC and HMBC, Methanol-*d*$_4$) δ 195.1 (C, C-10), 175.1 (C, C-11), 162.6 (C, C-1), 151.3 (C, C-5), 147.4 (C, C-2), 144.9 (C, C-4), 142.6 (C, C-6), 141.0 (C, C-3), 116.1 (C, C-3a), 114.0 (C, C-7a), 109.2 (CH, C-7), 52.8 (CH_3, 11-OMe), 42.1 (CH, C-8), 38.4 (CH_2, C-9); HR-ESI-MS (TOF) *m/z* $[M-H]^-$ 305.0325 (calc for $C_{14}H_9O_8^-$, 305.0297); MS^2-fragmentation (CE = −40 V) *m/z* 273 (12), 245 (53), 229 (5), 217 (100), 201 (9), 189 (23), 173 (9), 161 (16), 145 (13), 133 (10), 117 (5), 105 (2). Data corresponds to literature [27,28].

Ellagic acid (**12**): yellow solid; ^{1}H NMR (500 MHz, DMSO-*d*$_6$) δ 7.31 (2H, *s*, H-5 + H-5’); ^{13}C NMR (obtained from HSQC and HMBC, DMSO-*d*$_6$) δ 159.5 (2C, C-7 + C-7’), 148.6 (2C, C-4 + C-4’), 140.7 (2C, C-3 + C-3’), 113.0 (2C, C-6 + C-6’), 112.5 (2C, C-1 + C-1’), 108.1 (2CH, C-5 + C-5’); HR-ESI-MS (TOF) *m/z* $[M-H]^-$ 300.9978 (calc for $C_{14}H_5O_8$, 300.9984). Data corresponds to literature [29,30].

7’’-*O*-Methyl flavogallonate (**13**): yellow amorphous solid; ^{1}H NMR (400 MHz, Methanol-*d*$_4$) δ 7.54 (1H, *s*, H-5), 7.22 (1H, *s*, H-6’’), 3.50 (3H, *s*, 7’’-OMe); ^{13}C NMR (obtained from HSQC and HMBC, Methanol-*d*$_4$) δ 169.1 (C, C-7’’), 161.9 (C, C-7), 149.4 (C, C-4), 145.4 (C, C-5’’), 140.8 (C, C-3), 114.5 (C, C-1), 111.8 (CH, C-5), 111.4 (CH, C-6’’), 52.2 (CH_3, 7’’-OMe); HR-ESI-MS (TOF) *m/z* $[M-H]^-$ 483.0197 (calc for $C_{22}H_{11}O_{13}^-$, 483.0200); MS^2-fragmentation (CE = −40 V) *m/z* 483 (2), 451 (100), 432 (15), 422 (6), 407 (8), 395 (8), 379 (7), 367 (7), 351 (5), 335 (3), 323 (3), 299 (3). Data corresponds to literature [31].

6-*O*-*trans*-*p*-Coumaroyl-D-glucopyranose (**14**): white amorphous solid; ^{1}H NMR (600 MHz, Methanol-*d*$_4$) δ 7.63 (1H, *d*, *J* = 15.8 Hz, H-7’), 7.45 (2H, *d*, *J* = 8.3 Hz, H-2’ + H-6’), 6.80 (2H, *d*, *J* = 8.3 Hz, H-3’ + H-5’), 6.33 (1H, *d*, *J* = 15.8 Hz, H-8’), 5.10 (1H, *d*, *J* = 3.9 Hz, H-1α), 4.50 (H, *d*, *J* = 8.1 Hz, H-1β), 4.46 (1H, *m*,, H-6a), 4.30 (1H, *dd*, *J* = 12.0, 5.9 Hz, H-6b), 3.54 (1H, *m*, H-5), 3.40 – 3.31 (3H, *m*, H-2 + H-3 + H-4); ^{13}C NMR (obtained from HSQC and HMBC, Methanol-*d*$_4$) δ 169.1 (C, C-9’), 161.1 (C, C-4’), 146.8 (CH, C-7’), 131.2 (2CH, C-2’ + C-6’), 127.3 (C, C-1), 116.9 (2CH, C-3’, C-5’), 114.9 (CH, C-8), 98.4 (CH, C-1β), 94.1 (CH, C-1α), 77.9 (CH, C-3), 75.4 (CH, C-5), 73.8 (CH, C-2), 71.9 (CH, C-4), 64.8 (CH_2, C-6); HR-ESI-MS (TOF) *m/z* $[M-H]^-$ 325.0936 (calc for $C_{15}H_{17}O_8^-$, 325.0923); MS^2-fragmentation (CE = −20 V) *m/z* 325 (28), 307 (5), 265 (16), 217 (6), 204 (20), 187 (100), 163 (48), 161 (7), 145 (100), 119 (10), 113 (10), 89 (4). Data corresponds to literature [32].

1-*O*-Galloyl-D-glucose (**15**): white solid; ^{1}H NMR (400 MHz, Methanol-*d*$_4$) δ 7.13 (2H, *s*, H-2’ + H-6’), 5.65 (1H, *d*, *J* = 8.0 Hz, H-1β), 5.65 (1H, *d*, *J* = 3.1 Hz, H-1α), 3.85 (1H, *dd*, *J* = 12.1, 1.8 Hz, H-6a), 3.70 (1H, *dd*, *J* = 12.1, 4.5 Hz, H-6b), 3.49 – 3.43 (2H, *overlaid m*, H2 + H-5), 3.42 – 3.37 (2H, *overlaid m*, H-3 + H-4); ^{13}C NMR (obtained from HSQC and HMBC, Methanol-*d*$_4$) δ 166.6 (C, C-7’), 145.9 (2C, C-3’ + C-5’), 139.8 (C, C-4’), 120.2 (C, C-1’), 109.8 (2CH, C-2’ + C-6’), 95.6 (CH, C-1), 78.4 (CH, C-3), 77.7 (CH, C-5), 73.7 (CH, C-2), 70.5 (CH, C-4), 61.9 (CH_2, C-6); HR-ESI-MS (TOF) *m/z* $[M-H]^-$ 331.0640 (calc for $C_{13}H_{15}O_{10}^-$, 331.0665); MS^2-fragmentation (CE = −50 V) *m/z* 331 (100), 271 (10), 211 (16), 169 (58), 151 (15), 125 (17), 123 (21). Data corresponds to literature [33].

3,5-Di-*O*-galloylshikimic acid (**16**): whitish solid; ^{1}H NMR (600 MHz, Methanol-*d₄*) δ 7.13 (2H, *s*, H-2' + H-6'), 7.08 (2H, *s*, H-2'' + H-6''), 6.73 (1H, *pst*, *J* = 1.8 Hz, H-2), 5.76 (1H, *tt*, *J* = 3.7, 1.8 Hz, H-3), 5.42 (1H, *dt*, *J* = 7.4, 5.1 Hz, H-5), 4.23 (1H, *dd*, *J* = 7.4, 4.0 Hz, H-4), 2.98 (1H, *ddt*, *J* = 18.6, 5.1, 2.0 Hz, H-6a), 2.48 (1H, *ddt*, *J* = 18.6, 5.1, 1.7 Hz, H-6b); ^{13}C NMR (obtained from HSQC and HMBC, Methanol-d_4) δ 167.5 (C, C-7'), 167.4 (C, C-7''), 146.3 (2C, C-3'' + C-5''), 146.2 (2C, C-2' + C-5'), 139.7 (C, C-4'), 139.6 (C, C-4''), 135.2 (C, C-1), 131.6 (CH, C-2), 121.2 (C, C-1''), 121.0 (C, C-1'), 110.2 (2CH, C-2' + C-6'), 110.1 (2CH, C-2'' + C-6''), 71.6 (CH, C-5), 70.8 (CH, C-3), 68.1 (CH, C-3), 29.6 (CH_2, C-6); HR-ESI-MS (TOF) *m/z* [M−H]$^{-}$ 477.0647 (calc for $C_{21}H_{17}O_{13}^{-}$, 477.0675); MS^2-fragmentation (CE = −45 V) *m/z* 477 (100), 307 (12), 289 (39), 263 (23), 169 (81), 137 (45), 125 (48), 124 (17), 93 (20). Data corresponds to literature [34].

Chebulanin (**17**): brown amorphous solid; ^{1}H NMR (600 MHz, Methanol-d_4) δ 7.45 (1H, *s*, H-2''), 7.13 (2H, *s*, H-2''' + H-6'''), 6.35 (1H, *d*, *J* = 2.8 Hz, H-1), 5.20 (1H, *dt*, *J* = 2.8, 1.8 Hz, H-2), 5.09 (1H, *dd*, *J* = 7.1, 1.5 Hz, H-3'), 4.80 (2H, *brs*, H-3 + H-4), 4.77 (1H, *d*, *J* = 7.1 Hz, H-2'), 4.31 (1H, *t*, *J* = 6.5 Hz, H-5), 4.06 (1H, *dd*, *J* = 11.2, 6.6 Hz, H-6a), 4.00 (1H, *dd*, *J* = 11.2, 6.5 Hz, H-6b), 3.81 (1H, *ddd*, *J* = 12.0, 3.3, 1.5 Hz, H-4'), 2.17 (1H, *dd*, *J* = 17.0, 3.3 Hz, H-5'a), 2.11 (1H, *dd*, *J* = 17.0, 12.0 Hz, H-5'b); ^{13}C NMR (obtained from HSQC and HMBC, Methanol-d_4) δ 175.1 (C, C-6'), 174.7 (C, C-7'), 170.8 (C, C-1'), 166.8 (C-7''), 166.4 (C, C-7'''), 147.4 (C, C-3''), 146.8 (2C, C-3''' + C-5'''), 141.5 (C, C-5''), 140.3 (C, C-4'''), 140.2 (C, C-4''), 120.7 (C, C-1'''), 119.5 (C, C-1''), 117.6 (CH, C-2''), 116.1 (C-6''), 110.4 (2CH, C-2''' + C-6'''), 93.2 (CH, C-1), 79.9 (CH, C-5), 74.3 (CH, C-2), 72.3 (CH, C-4), 67.3 (CH, C-2'), 63.8 (CH_2, C-6), 62.1 (CH, C-3), 41.8 (CH, C-3'), 40.2 (CH, C-4'), 31.0 (CH_2, C-5'); HR-ESI-MS (TOF) *m/z* [M−H]$^{-}$ 651.0837 (calc for $C_{27}H_{23}O_{19}^{-}$, 651.0834); MS^2-fragmentation (CE = −45 V) *m/z* 651 (58), 633 (12), 481 (28), 463 (9), 453 (10), 437 (10), 49 (10), 381 (12), 331 (9), 319 (11), 293 (11), 275 (23), 247 (12), 231 (24), 205 (17), 203 (16), 193 (11), 175 (9), 169 (100), 125 (24). Data corresponds to literature [35].

Chebulagic acid (**18**): brown amorphous solid; ^{1}H NMR (500 MHz, Methanol-d_4) δ 7.48 (1H, s, H-2''), 7.07 (2H, s, H-2''' + H-6'''), 6.84 (1H, *s*, 3,6-HHDP-H-5), 6.63 (1H, *s*, 3,6-HHDP-H-5'), 6.50 (1H, *psd t*, *J* = 1.2 Hz, H-1), 5.82 (1H, *td*, *J* = 0.9, 2.3 Hz, H-3), 5.39 (1H, *brs*, H-2), 5.22 (1H, *psd d*, *J* = 3.2 Hz, H-4), 5.04 (1H, *dd*, *J* = 7.1, 1.4 Hz, H-3'), 4.90 (1H, *overlaid m*, H-6b), 4.83 (1H, *overlaid m*, H-5), 4.80 (1H, *d*, *J* = 7.1 Hz, H-2'), 4.37 (1H, *dd*, *J* = 10.4, 7.8 Hz, H-6a), 3.79 (1H, *dd*, *J* = 11.8, 3.7 Hz, H-4'), 2.19 (1H, *dd*, *J* = 17.0, 3.7 Hz, H-5'b), 2.11 (1H, *dd*, *J* = 17.0, 11.8 Hz, H-5'a); ^{13}C NMR (126 MHz, Methanol-d_4) δ 175.1 (C, C-6'), 174.4 (C, C-7'), 170.8 (C, C-1'), 170.1 (C, 3,6-HHDP-C-7'), 167.5 (C, 3,6-HHDP-C-7), 166.4 (C-7''), 166.3 (C, C-7'''), 147.4 (C, C-3''), 146.5 (2C, C-3''' + C-5'''), 146.1 (C, 3,6-HHDP-C-4'), 145.6 (C, 3,6-HHDP-C-4), 145.5 (C, 3,6-HHDP-C-2), 145.3 (C, 3,6-HHDP-C-2'), 141.4 (C, C-5''), 140.8 (C, C-4'''), 140.4 (C, C-4''), 138.7 (C, 3,6-HHDP-C-3), 137.6 (C, 3,6-HHDP-C-3'), 125.6 (C, 3,6-HHDP-C-6'), 124.5 (C, 3,6-HHDP-C-6), 120.1 (C, C-1'''), 119.0 (C, C-1''), 117.6 (1CH + 1C, C-2'' + 3,6-HHDP-C-1), 116.2 (C, C-3,6-HHDP-C-1'), 116.0 (C-6''), 110.9 (2CH, C-2''' + C-6'''), 110.4 (CH, 3,6-HHDP-C-5), 108.2 (CH, 3,6-HHDP-C-5'), 92.5 (CH, C-1), 74.3 (CH, C-5), 71.1 (CH, C-2), 67.0 (C, C-2'), 66.8 (CH, C-4), 64.7 (CH_2, C-6), 62.4 (CH, C-3), 41.7 (CH, C-3'), 40.0 (CH, C-4'), 30.6 (CH_2, C-5'); HR-ESI-MS (TOF) *m/z* [M−H]$^{-}$ 953.0830 (calc for $C_{41}H_{29}O_{27}^{-}$, 953.0896); MS^2-fragmentation (CE = −50 V) *m/z* 953 (41), 935 (4), 783 (2), 633 (5), 615 (3), 481 (6), 463 (5), 337 (10), 319 (8), 301 (100), 275 (16), 249 (3), 231 (3), 205 (8), 169 (4). Data corresponds to literature [35].

6’-*O*-Methyl-chebulagic acid (**19**): brown amorphous solid; ^{1}H NMR (400 MHz, Methanol-d_4) δ 7.47 (1H, *s*, H-2’’), 7.07 (2H, *s*, H-2’’’ + H-6’’’), 6.84 (1H, *s*, 3,6-HHDP-H-5), 6.64 (1H, *s*, 3,6-HHDP-H-5’), 6.50 (1H, *brs*, H-1), 5.76 (1H, *td*, *J* = 0.9, 2.4 Hz, H-3), 5.39 (1H, *brs*, H-2), 5.23 (1H, *psd d*, *J* = 3.5 Hz, H-4), 5.06 (1H, *dd*, *J* = 7.2, 1.2 Hz, H-3’), 4.93 (1H, *d*, *J* = 10.7 Hz, H-6b), 4.83 (1H, *overlaid m*, H-5), 4.81 (1H, *d*, *J* = 7.2 Hz, H-2’), 4.37 (1H, *dd*, *J* = 10.7, 7.8 Hz, H-6a), 3.83 (1H, *ddd*, *J* = 11.6, 3.7, 1.2 Hz, H-4’), 3.58 (3H, *s*, H-6’OMe), 2.20 (1H, *dd*, *J* = 16.7, 3.9 Hz, H-5’b), 2.12 (1H, *dd*, *J* = 17.0, 11.6 Hz, H-5’a); ^{13}C NMR (100 MHz, Methanol-d_4) δ 174.3 (C, C-7’), 173.6 (C, C-7’), 170.6 (C, C-1’), 170.1 (C, 3,6-HHDP-C-7’), 167.5 (C, 3,6-HHDP-C-7), 166.3 (C-7’’), 166.2 (C, C-7’’’), 147.5 (C, C-3’’), 146.5 (2C, C-3’’’ + C-5’’’), 146.2 (C, 3,6-HHDP-C-4’), 145.6 (C, 3,6-HHDP-C-4), 145.5 (C, 3,6-HHDP-C-2), 145.4 (C, 3,6-HHDP-C-2’), 141.4 (C, C-5’’), 140.9 (C, C-4’’’), 140.4 (C, C-4’’), 138.7 (C, 3,6-HHDP-C-3), 137.6 (C, 3,6-HHDP-C-3’), 125.6 (C, 3,6-HHDP-C-6’), 124.5 (C, 3,6-HHDP-C-6), 120.1 (C, C-1’’’), 119.0 (C, C-1’’), 117.6 (1CH + 1C, C-2’’ + 3,6-HHDP-C-1), 116.2 (C, C-3,6-HHDP-C-1’), 115.8 (C-6’’), 110.9 (2CH, C-2’’’ + C-6’’’), 110.5 (CH, 3,6-HHDP-C-5), 108.2 (CH, 3,6-HHDP-C-5’), 92.4 (CH, C-1), 74.1 (CH, C-5), 71.0 (CH, C-2), 67.0 (C, C-2’), 66.8 (CH, C-4), 64.7 (CH_2, C-6), 62.4 (CH, C-3), 52.5 (CH3, C-6’OMe), 41.8 (CH, C-3’), 39.9 (CH, C-4’), 30.5 (CH_2, C-5’); HR-ESI-MS (TOF) *m/z* $[M-H]^-$ 967.1026 (calc for $C_{42}H_{31}O_{27}^-$, 953.1053); MS^2-fragmentation (CE = −45 V) *m/z* 967 (100), 923 (18), 797 (5), 301 (62), 275 (10), 237 (7), 205 (8), 169 (5). Data corresponds to literature [25].

Phyllanembilinin C (**20**): yellow solid;^{1}H NMR (600 MHz, Methanol-d_4) δ 7.05 (2H, *s*, H-2’’’ + H-6’’’), 7.01 (1H, *s*, H-2’’), 6.81 (1H, *s*, 3,6-HHDP-H-5), 6.63 (1H, *s*, 3,6-HHDP-H-5’), 6.49 (1H, *s*, H-1), 5.93 (1H, *m*, H-3), 5.26 (1H, *brs*, H-2), 5.17 (1H, *d*, *J* = 3.5 Hz, H-4), 4.94 (1H, *q*, *J* = 10.6 Hz, H-6b), 4.80 (1H, *overlaid m*, H-5), 4.72 (1H, *d*, *J* = 2.2 Hz, H-3’), 4.34 (1H, *dd*, *J* = 10.6, 8.2 Hz, H-6a), 3.70 (1H, *dt*, *J* = 11.7, 2.2 Hz, H-4’), 2.67 (1H, *dd*, *J* = 17.4, 2.1 Hz, H-5’b), 1.93 (1H, *dd*, *J* = 17.4, 11.7 Hz, H-5’a); ^{13}C NMR (obtained from HSQC and HMBC, Methanol-d_4) δ 176.4 (C, C-6’), 174.9 (C, C-7’), 174.8 (C, C-1’), 170.2 (C, 3,6-HHDP-C-7'), 167.7 (C, 3,6-HHDP-C-7), 166.5 (C, C-7’’), 166.2 (C, C-7’’’), 148.0 (C, C-5’’), 147.8 (C, C-3’’), 146.6 (2C, C-3’’’ + C-5’’’), 146.3 + 146.0 (2C, 3,6-HHDP-C-2' + 3,6-HHDP-C-4'), 145.8 + 145.5 (2C, 3,6-HHDP-C-2 + 3,6-HHDP-C-4), 140.7 (C, C-4’’’), 138.6 (C, 3,6-HHDP-C-3), 137.6 (C, 3,6-HHDP-C-3’), 136.1 (C, C-4’’), 125.6 (C, 3,6-HHDP-C-6’), 124.7 (C, 3,6-HHDP-C-6), 122.2 (C, C-1’’), 120.4 (C, C-1’’’), 117.6 (C, 3,6-HHDP-C-1), 117.4 (C, C-6’’), 116.4 (C, 3,6-HHDP-C-1’), 112.2 (CH, C-2’’), 111.0 (2CH. C-2’’’ + C-6’’’), 110.0 (CH, 3,6-HHDP-C-5), 108.2 (CH, 3,6-HHDP-C-5‘), 93.0 (CH, C-1), 74.1 (CH, C-5), 70.1 (CH, C-2), 66.9 (CH, C-4), 64.9 (CH_2, C-6), 62.1 (CH, C-3), 50.1 (CH, C-3’), 42.6 (CH, C-4’), 31.6 (CH_2, C-5’); HR-ESI-MS (TOF) *m/z* $[M-H]^-$ 969.0831 (calc for $C_{41}H_{29}O_{28}^-$, 969.0845); MS^2-fragmentation (CE = −45 V) *m/z* 969 (100), 925 (4), 711 (2), 633 (2), 463 (3), 301 (45), 275 (4), 247 (17), 203 (6), 175 (2). Data corresponds to literature [36].
